# Long Intergenic Non-Coding RNAs: Novel Drivers of Human Lymphocyte Differentiation

**DOI:** 10.3389/fimmu.2015.00175

**Published:** 2015-04-15

**Authors:** Ilaria Panzeri, Grazisa Rossetti, Sergio Abrignani, Massimiliano Pagani

**Affiliations:** ^1^Integrative Biology Unit, Istituto Nazionale Genetica Molecolare “Romeo ed Enrica Invernizzi”, IRCCS Ospedale Maggiore Policlinico, Milano, Italy; ^2^Department of Medical Biotechnology and Translational Medicine, Università degli Studi di Milano, Milano, Italy

**Keywords:** long non-coding RNAs, epigenetic regulation, lymphocyte differentiation

## Abstract

Upon recognition of a foreign antigen, CD4^+^ naïve T lymphocytes proliferate and differentiate into subsets with distinct functions. This process is fundamental for the effective immune system function, as CD4^+^ T cells orchestrate both the innate and adaptive immune response. Traditionally, this differentiation event has been regarded as the acquisition of an irreversible cell fate so that memory and effector CD4^+^ T subsets were considered terminally differentiated cells or lineages. Consequently, these lineages are conventionally defined thanks to their prototypical set of cytokines and transcription factors. However, recent findings suggest that CD4^+^ T lymphocytes possess a remarkable phenotypic plasticity, as they can often re-direct their functional program depending on the milieu they encounter. Therefore, new questions are now compelling such as which are the molecular determinants underlying plasticity and stability and how the balance between these two opposite forces drives the cell fate. As already mentioned, in some cases, the mere expression of cytokines and master regulators could not fully explain lymphocytes plasticity. We should consider other layers of regulation, including epigenetic factors such as the modulation of chromatin state or the transcription of non-coding RNAs, whose high cell-specificity give a hint on their involvement in cell fate determination. In this review, we will focus on the recent advances in understanding CD4^+^ T lymphocytes subsets specification from an epigenetic point of view. In particular, we will emphasize the emerging importance of non-coding RNAs as key players in these differentiation events. We will also present here new data from our laboratory highlighting the contribution of long non-coding RNAs in driving human CD4^+^ T lymphocytes differentiation.

## The Revolutions of Regulatory Non-Coding RNAs

At the beginning of this century, the results of the human genome project highlighted the complexity of our genome. What emerged was that the fraction of the genome that is informative is higher than we expected. Subsequent analysis revealed that the vast majority of informative sequences does not encode for proteins. Indeed against a total of 62.1% of the human genome covered by processed transcript (74.7% by primary transcripts), exons of protein-coding genes cover only the 2.94% of the genome (1). From an evolutionary point of view, the genome size is in close relationship with coding potential in prokaryotes, which have haploid genomes primarily composed by protein-coding sequences (~88%). Conversely, in eukaryotes, a correlation lacks between protein-coding gene number and organismal complexity. These observations are likely explained by the evolution of a more sophisticated architecture to control gene expression that includes the expansion of non-coding regulatory RNAs (ncRNAs) (2). Thus, we should clearly reassess the centrality of protein-coding RNAs in favor of non-coding ones.

Non-coding RNAs with fundamental functions within cells are known since the discovery of the first transfer RNA (tRNA) (3) and comprise also ribosomal RNAs (rRNAs). Nonetheless, the interest toward non-coding RNAs with regulatory functions arose with the discovery of the first human micro-RNA, let-7 (4). In order to apply a theoretical framework to the transcriptome, regulatory ncRNAs are usually classified based on their dimension: “small” ncRNAs being less than 200 nucleotides in length and “long” or “large” ncRNAs (lncRNAs) ranging from more than 200 to tens of thousands of nucleotides (Table 1).

**Table 1:** **Major classes of short and long regulatory non-coding RNAs**.

ncRNA		Length (nt)	Function
**SHORT**			
miRNAs	Micro RNAs	21–23	In animals, associate with the miRNA-induced silencing complex (RISC) and silence the expression of target genes mostly post-transcriptionally (5–7)
snoRNAs	Small nucleolar RNAs	60–300	Help the chemical modification of mRNAs, thereby influencing stability, folding, and protein-interaction properties (8, 9)
snRNAs	Small nuclear RNAs	150	Assist splicing of introns from primary genomic transcripts (10, 11)
piRNAs	Piwi-interacting RNAs	25–33	Associate with the highly conserved Piwi family of argonaute proteins and are essential for retrotransposon silencing in germline, epigenetic modifications, DNA rearrangements, mRNA turnover, and translational control also in soma (12–14)
PASRs	Promoter-associated short RNAs	22–200	Enriched at the 5′end of genes, within 0.5 kb of TSS. Can be transcribed both sense and antisense. Their function and biogenesis is not fully understood (15, 16)
TASRs	Termini-associated short RNAs	22–200	Can be transcribed both sense and antisense near termination sites of protein-coding genes. Their function and biogenesis is not fully understood (15, 16)
siRNAs	Short interfering RNAs	21–23	Processed from a plethora of genomic sources, both foreign (viruses) and endogenous (repetitive sequences). Canonically induce the degradation of perfectly complementary target RNAs (17, 18)
tiRNAs	Transcription initiation RNAs	15–30	Enriched immediately downstream transcriptional start sites (TSSs) of highly expressed genes. Their function and biogenesis is not fully understood (16, 19, 20)
**LONG**			
NATs	Natural antisense transcripts	>200	Transcribed from the same locus but opposite strand of the overlapping protein-coding sequence. Involved in gene expression regulation, RNA editing, stability, and translation (21, 22)
PALRs	Promoter-associated long RNAs	200–1000	Enriched at promoters, found to regulate gene expression (23, 24)
PROMPTs	Promoter upstream transcripts	200–600	Enriched at TATA-less, CpG-rich promoters with broad TSSs. Affect promoter methylation and regulate transcription (25–27)
T-UCRs	Transcribed ultraconserved regions	>200	Perfectly conserved between human, rat, and mouse. Frequently located at fragile sites and at genomic regions involved in cancer (28)
Intronic RNAs		>200	Transcribed from introns of overlapping protein-coding sequences. Involved in the control of gene expression, alternative splicing, and source for generation of shorter regulatory RNAs (29)
eRNAs	Enhancer-derived RNAs	>200	Function still not completely understood. May functionally contribute to the enhancer function (30–32)
LincRNAs	Long intervening (intergenic) RNAs	>200	Gene expression regulation, regulation of cellular processes (33, 34)
uaRNAs	3′UTR-derived RNAs	<1000	Derive within 3′untranslated region (3′UTR) sequences. Function still not clearly understood (35)
circRNA	Circular RNA	100 to >4000	Diverse, from templates for viral replication to transcriptional regulators (36)

Further complicating the picture, lncRNAs seem to be the preferred substrate for the generation of small RNAs (21). This maze of non-coding transcripts was revealed also in a genome-wide identification of lncRNAs in mouse CD8^+^ T lymphocytes, where 18 of the identified lncRNAs appeared to overlap with annotated miRNAs and 21 with snoRNAs (37).

Both classes can be further classified according to their position relative to known sequences of the genome, like in the case of promoter-associated RNAs (PASRs) or transcription initiation small RNAs (tiRNAs). In particular, long non-coding RNAs are usually classified relative to neighboring protein-coding genes. They can be defined as “sense” if they are transcribed from the same strand of the protein-coding gene or “antisense” if the opposite is true. They can be “divergent” if their promoter and the one of the coding transcript are in close proximity and located in a head to head fashion. They can be “exonic” or “intronic” if they overlap one or more exons, or an intron of the protein-coding gene respectively. Instead, they can be “intergenic” (or “intervening”; lincRNAs) if they lie within a sequence between two protein-coding genes (38). In this review, we will focus on this last category, which is probably the most studied given that the location of these lncRNAs avoids complications deriving from the overlap with other genes. The majority of known lncRNAs is generated by the same transcriptional machinery of mRNAs. This means that transcribed lincRNAs genomic sequences are marked by RNA polymerase II occupancy and histone modifications that are shared with active protein-coding genes, such as H3K4me3 at promoters and H3K36me3 within gene bodies (39). They are capped by methylguanosine at their 5′, spliced, and polyadenylated, even if the widespread representation of this last property among known lncRNAs could be partially due to the RNA sequencing strategies used for their identification (15, 40). Indeed, broader analysis identified about 39% of lncRNAs to have at least one of the six most common poly(A) motifs, compared to 51% for coding transcripts (1). These properties imply that there are few distinctive biochemical features that allow the distinction of lncRNAs from protein-coding mRNAs. Among them, lncRNAs have unusual exon structure, with on average 2–5 exons. Intriguingly, lncRNAs are significantly more likely to overlap repetitive elements and particularly RNA-derived transposable elements (TEs). These last account for about 30% of human lncRNAs nucleotides, often in proximity of their transcriptional start site (TSS), which could suggest that TEs could be important drivers of lncRNAs evolution (see below). Nonetheless, the main difference between lncRNAs and protein-coding genes relies by definition on their coding potential: lncRNAs does not possess open reading frames (ORFs), as evaluated based on: the conservation of ORFs codons (41), ORFs length, the presence of known protein domains, *in vitro* translation (42, 43), and ribosome footprinting (44) assays. However, these conceptual constraints are terribly artificial: short, non-canonical peptides have been found to arise from small ORFs within ncRNA (45–48); lncRNAs genes can also code for proteins and have a double function (49) and ultimately, the coding potential does not necessarily exclude a function as RNA also for known mRNAs (50). Evolution makes boundaries between coding and non-coding genes fainter as ncRNAs can evolve by pseudogenization. This event can follow disruption of the ancestral ORF, but not of the untranslated regulatory regions (UTRs) in protein-coding genes duplicates (50) or can arise without duplication, but from the co-option of ancestral genes to different, non-coding functions (51). This was the case of the long-known Xist RNA, involved in the silencing of the inactive X chromosomes in eutherians. In particular, two exons of the protein-coding gene Lnx3 are homologous to Xist. This gene retained a protein-coding capacity at least in the common ancestor of marsupials and placentals. Conversely, the Xist A-repeat implicated in X-silencing function is not conserved. This sequence likely arose from the insertion of a TE recruited to form a proto-Xist gene (52, 53). Therefore, the difference between dosage compensation in marsupials, eutherians, and monotremes can be ascribed from the presence of a Xist-independent XCI in mammalian ancestor and the peculiar evolution of the proto-Xist gene by pseudogenization in the eutherian ancestor. Intriguingly, other lncRNAs involved in X-inactivation are similarly examples of pseudogenization (54). The boundary between coding and non-coding is even less defined when ncRNAs arise from joining of coding and non-coding exons through alternative splicing (55, 56), from untranslated regions of mRNAs (57, 58) or from the opposite strand of the overlapping protein-coding gene (59). Strikingly, more than a half of protein-coding genes in mammals have a complementary non-coding transcript (60). These findings further challenge our “linear” model of the genome, prompting a re-evaluation of current dogma and genes definitions. Genomic regions indeed are far more complex than previously thought: genes can be used for different purposes and different functional elements can co-locate intermingling coding and non-coding regions.

The interest toward lncRNAs has been rapidly growing and their expressions have been quantitated in many different tissues and cell types by high-throughput sequencing (RNA-seq). These efforts retrieved catalogs with little overlap, so that the number of known lncRNAs is still growing, in contrast with the number of known protein-coding genes that has been remarkably stable over years. Indeed, lncRNAs are far more cell-specific than mRNAs, generally less but also more dynamically expressed at various differentiation stages. For this reason, immune system is an excellent context in which we can deepen our knowledge on lncRNAs. While many excellent reviews cover the recent advances in understanding the role of these molecules within the innate branch (61, 62), little is still known about their importance for the human adaptive immune system. Effector lymphocytes are highly specialized cells that arise from common progenitors through differentiation processes still not completely understood. Besides, lymphocytes can be purified through cell sorting from blood of healthy donors and the existence of *in vitro* differentiation protocols provide the ideal setting for the identification of lncRNAs expressed in the human immune system and for their functional characterization.

Indeed, the growing interest on lncRNAs and the lack of knowledge on their expression patterns in the human immune system prompted us to perform the RNA-seq analysis on 13 human primary lymphocytes subsets purified by FACS sorting from healthy donors (CD4^+^ naïve, T_H_1, T_H_2, T_H_17, T_reg_, T_CM_, T_EM_, CD8^+^ naïve, T_CM_, T_EM_, B naïve, B memory, B CD25^+^) and to develop a bioinformatics pipeline for lincRNAs identification.

Through this analysis, we identified long intergenic ncRNAs genes expressed in these subsets and confirmed that lincRNAs cell-specificity is higher than protein-coding genes even when comparing lincRNAs genes with membrane receptor protein-coding genes, which are generally referred as the most accurate markers for lymphocyte subsets definition. Besides, a major outcome of this analysis is the identification through *de novo* transcriptome reconstruction of 563 novel, previously unannotated long intergenic ncRNAs genes, increasing by ~12% the number of lincRNAs known to be expressed in human lymphocytes (63). Intriguingly, a fraction of lincRNAs specific for B cells and a fraction of “pan-T” lincRNAs also exist (63). It would be extremely interesting to study these lincRNAs during lymphocytes development in order to understand their likely peculiar role in thymic or bone-marrow-derived cells development.

These observations imply that the little overlap between available catalogs is a direct consequence of lncRNAs specificity and that we could overcome this limitation only assessing lncRNAs expression in every different, highly purified cell type at different developmental stages, instead of considering tissues as a whole. Moreover, due to their specificity of expression, human lymphocytes lincRNAs that are not yet annotated in public resources would have not been identified without performing *de novo* transcriptome reconstruction. As mentioned before, such tissue-specificity has been linked to the enrichment of TEs in proximity to lincRNAs TSS (64, 65). Moreover, RNA-seq experiments performed in a human CD4^+^ naïve T cells *in vitro* differentiation time-course suggest that lincRNA-specific expression in human lymphocyte subsets is acquired during their activation-driven differentiation from naïve to memory cells (63).

These findings hint to the involvement of lymphocyte-specific lincRNAs as fine-tuners in cell fate commitment, differentiation, and maintenance of cell identity, as demonstrated by many examples in other cell types (66–68). Also, lincRNAs are functionally involved in cell growth (69, 70), apoptosis (71–74), development (75–77), imprinting (78–80), and dosage compensation (81) in almost every cellular context (Table 2).

**Table 2:** **Examples of lincRNAs with key roles in various cellular contexts**.

LncRNA	Cellular context	Function
H19	HSC, placenta	Maternally expressed imprinted gene important for inhibiting placental *growth* (69) and maintaining adult hematopoietic stem cell populations (HSC) via miR-675 generation and repression of Igf1r (70)
GAS5	T lymphocytes, cancers	Plays an essential role in normal *growth arrest* in T lymphocytes (71). Its increased level of expression correlate with cell death and reduced cell proliferation both in prostate (72) and colorectal cancer cell lines. Its lower expression is instead significantly correlated with larger tumor size and poor prognosis in colorectal cancer patients (74)
Linc-MD1	Muscles	Governs the timing of muscle *differentiation* by acting as a competing endogenous RNA (ceRNA) with respect to miR-133 and miR-135 in mouse and human myoblasts (75)
Xist	Somatic cells	Expressed by the future inactive X chromosome, triggers gene silencing *in cis* by coating the chromosome. It induces a cascade of chromatin changes, post-translational histone modification and DNA methylation, and leads to the stable repression of X-linked genes, ensuring proper *dosage compensation* (81)
KCNQ1OT1	Most tissues	Paternally expressed antisense transcript to an interior portion of *Kncq1*, part of an imprinted locus on human chromosome 11p15. It is critical for *imprinting*-mediated silencing in most tissue, via long-range intrachromosomal loop and recruitment of polycomb repressive complex 2 (PRC2) (78–80)

**Figure 1:**
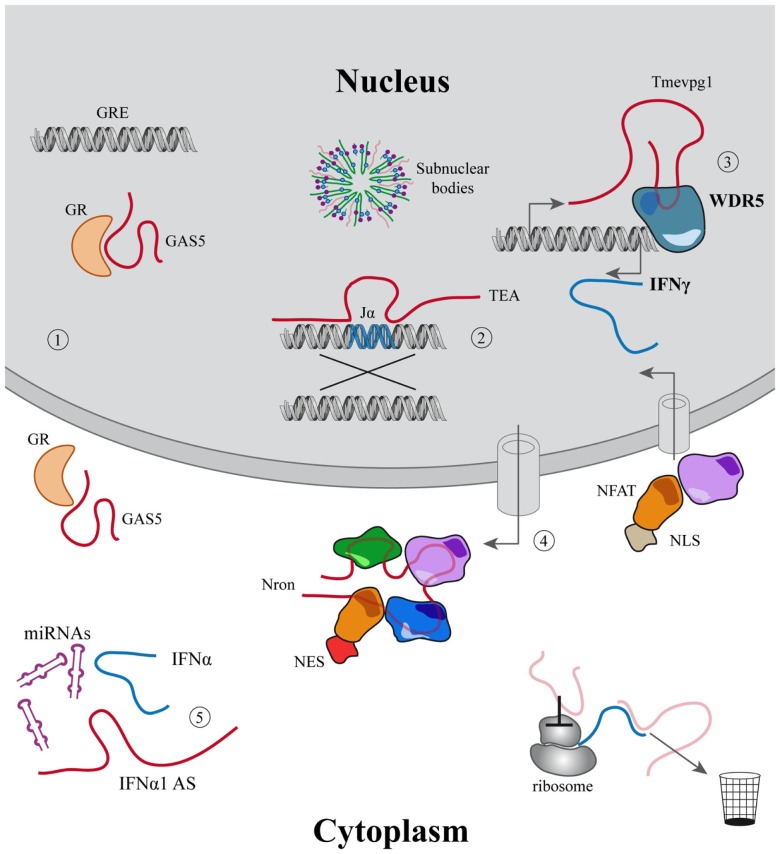
**Examples of the main functions associated to lincRNAs**. (1–5) LincRNAs described in the immune system. (1) Modulation of cell growth and apoptosis mediated by GAS5 that acts inhibiting glucocorticoid receptors binding to their DNA responsive elements; (2) Jα recombination guided by the PARL TEA; (3) Tmevpg1 recruits WDR5 to induce IFNγ expression; (4) Nron modulates the import–export of NFAT to the nucleus; (5) IFNα1-AS acts as a competing endogenous RNA, releasing IFNα from micro-RNA inhibition. In light red other mechanisms are described for lincRNAs outside the immune system.

Long non-coding regulatory RNAs exert their function in these fundamental processes interacting with chromatin or DNA modifiers and transcription factors (TFs) modulating gene expression (82); competing with micro-RNAs acting as sponges (83); modulating subcellular trafficking (84), translation (85), splicing (86), and likely through many other mechanisms still to be discovered (Figure 1).

Long non-coding regulatory RNAs functional flexibility derive from their intrinsic propensity to fold into thermodynamically stable secondary and higher orders structures that function as interaction modules (87). Each module can fold independently from another, forming bonds at the level of Watson–Crick, Hoogstein, and ribose face (88, 89). These RNAs can rapidly shift between diverse stable structural conformation, allowing allosteric transitions that can act as switches in response to environmental stimuli. They are also processed faster than mRNA, given that they must not be translated, allowing a rapid response to signals. LncRNAs can also be regulated via more than a hundred different nucleotide modifications, like in the case of tRNAs, rRNAs, and snoRNAs (90–92) that modulate their function and probably their structure. RNAs can generate multiple modules within their structure, allowing the interaction with multiple players, the reception of multiple stimuli, and the generation of multiple outputs. The required pairing is likely extremely flexible, such as in the case of micro-RNAs, and allows mismatches, bulges, and wobblings (93). Many of these interaction modules derive from repetitive elements, such as transposons that took advantage of the fewer constraints that lncRNAs sequences have compared to protein-coding genes (1, 94). Indeed, lncRNAs rate of sequence evolution is higher relative to protein-coding genes, even if also these transcripts exhibit evolutionary signatures of functionality. They evolve under modest but detectable selective pressure, accumulating fewer substitutions than neutrally evolving sequences (95, 96). Likely, conservation of relatively small units of lncRNAs sequences (estimated to be less than 5%) could be sufficient to preserve their function, considering their already mentioned modular structure (97). This could be the reason why existing bioinformatic approaches fail to detect low level and scattered selective constraint within these loci (97).

Through such a plastic and versatile structure, lncRNAs can exert their functions binding to proteins, other RNAs (98), and probably also DNA, even if there is still little evidence on the existence of RNA:DNA triplex (99, 100). In particular, lncRNAs can act as scaffolds, bridging together different molecules in a coordinated hub, like in the case of NEAT1: a highly abundant lncRNA that controls sequestration of proteins involved in the formation of paraspeckles, nuclear domains associated with mRNA retention and pathologically enriched in influenza and herpes viruses infections (101, 102). LncRNA can also act as guides, recruiting proteins at specific loci: this has been hypothesized in the case of recombination events that mediate genetic diversity in developing lymphocytes as class switch (CS) and V(D)J recombinations that seem to be mediated by sense and antisense transcripts that dictates the locations of combinatorial events (103–105). Again, lncRNAs can act as control devices or riboswitches in response to extracellular stimuli. For example, they can act as decoys, precluding pre-existing interactions such as GAS5 RNA that detach glucocorticoid receptor (GR) from its responsive elements in conditions of growth arrest (106, 107). Nonetheless, the regulatory potential of lncRNAs has been better characterized in the context of the epigenetic regulation of transcription that ultimately defines the cell transcriptome.

## The Role of Long Non-Coding RNAs in Epigenetics

Histones and DNA modifications together with the tridimensional chromosomes conformation within the nucleus define, at least in part, the epigenetic landscape of the cell. This extremely dynamic context modulates gene expression and dictates the final transcriptional output in response to environmental stimuli. By definition, these modifications are then propagated throughout cell divisions. This process is important in every moment of cell life, but particularly during differentiation. Indeed, every cell within our body harbor the same genome, but every cell acquires a particular phenotype according to intrinsic and extrinsic cues that ultimately defines its epigenome and therefore its fate during differentiation. Epigenetics also defines to what extent this fate can be irreversible or plastic (108–110).

As mentioned before, human lymphocytes are an interesting model system for understanding the basis of cell fate specification and plasticity. Indeed, although traditionally the broad range of effector lymphocytes has been referred to as composed by distinct lineages, it has become increasingly clear that these cells also have notable features of plasticity. Differentiation of naïve cells into specific helper subsets requires the integration of extrinsic cues that converge into cell-intrinsic changes in the epigenetic landscape on the genome (111, 112). The interest within the field has been focused on the regulation of prototypical cytokine genes for each subset such as *Ifng* gene for T_H_1 or *Il-4* for T_H_2 CD4^+^ lymphocytes. Much work has been done in both cases to define the complex genetic structure of these loci and the *cis* regulatory elements bound by TFs and chromatin modifiers promoting or repressing their transcription (113–115). The importance of the setting of epigenetic memory at these fundamental loci was underlined also by treatment with DNA methylation inhibitors (116, 117) or histones deacetylases inhibitors (118–120) and by deletion of DNA methyltransferase (121–123), which caused respectively: constitutive production of IFN-γ, enhanced production of both T_H_1 and T_H_2 prototypic cytokines, and inability to activate the proper pattern of expressed cytokines. The same is true for deletion of components of trithorax group (TrxG) or polycomb repressive complex (PRC) that dictates active or repressive epigenetic marks at fundamental loci for proper T-helper cell differentiation, such as *Il-4*, *Il-5*, *Il-13*, and *Gata3* (124–129). The pattern of chromatin marks is conventional for signature cytokines: active marks are present at prototypical cytokines whereas repressive marks restrain the expression of antagonistic molecules. However, master regulators and other TFs, usually considered as definers of lineage-specific identity, are characterized by bivalent poised domains, in which both active and repressive chromatin marks are present (130, 131). This histone epigenetic status is peculiar also to promoters in embryonic stem cells, where it poises the expression of key developmental genes thus allowing their timely activation in the presence of differentiation signals and concomitantly precluding expression in their absence (132). Indeed, while the expression of master TFs is quite rapid, cell divisions are required for cytokine loci to become accessible or conversely repressed. Indeed, GATA3 and T-bet/STAT proteins initiate the epigenetic changes at IFN-γ and IL-4 loci that follow the initial activation of naïve T cells and differentiation toward T_H_1 and T_H_2 cell fate (133, 134). These observations imply that T-helper cells harbor both clear-cut and plastic epigenetic marks. Nonetheless, we must consider that even cytokines genes that are clearly defined epigenetically, can be expressed or repressed in unexpected context, as reported in T_H_1 cells converted in IL-4-producing cells during strong T_H_2-polarizing helminth infections (135) or stable T_H_1/T_H_2 hybrid cells derived after parasite infections (136). Therefore, other players must be involved to define the degree of plasticity of lymphocytes in response to these ever-changing environmental conditions during differentiation.

Long non-coding regulatory RNAs have been linked to epigenetic control of gene expression since the first studies regarding the already mentioned Xist transcript, involved in X chromosome inactivation in eutherians. Many other lncRNAs have been associated to chromatin or DNA modifiers and even TFs, thanks to specific mechanistic studies or high-throughput screenings (82, 137–139). This interplay can be observed across a broad range of eukaryotic organisms, suggesting that the epigenetic role of lncRNAs is conserved, even if their mere sequence conservation is often limited (as described previously). It seems that lncRNAs could act as scaffolds, physically associating with proteins that modify chromatin either activating or repressing gene expression. Thanks to the already discussed structural properties of RNA, lncRNAs could organize multiple players in spatially and temporally concerted actions (138). Not only: thanks to their ability to base pair with other nucleic acids, they could recruit these modifiers at specific loci, therefore conferring them specificity of action (98). This property has been an unsolved issue, given that chromatin modifiers do not possess intrinsic bias toward consensus sequences, at least in mammals, while in *Drosophila* these “docking sites” are well defined (140, 141). Interestingly, while many of these enzymes lack DNA-binding properties, they instead possess RNA-binding motifs (142–144).

The majority of reported lncRNAs are involved in the repression of gene transcription, in particular by interacting with polycomb group (PcG) proteins. The first examples of a direct interaction with PRC2 are the already mentioned Xist (145) and Kcnq1ot1, expressed only in the mammalian paternal chromosome and involved in the silencing of 8–10 protein-coding genes (146). In both these cases, lncRNAs are strictly required for the enrichment of PRC2-associated proteins and for the trimethylation of the lysine 27 of histone H3 at specific loci. Furthermore, other lncRNAs such as NEAT2 and TUG1 promote relocation of growth-control genes at foci of PcG proteins (called PcG bodies), therefore likely facilitating the concerted repression/activation of the transcription units in response to mitogenic signal (147). Many other protein complexes have been found to interact with lncRNAs, the majority targeting histones, either methylases or demethylases, but other involved in DNA methylation (148). Indeed, lncRNAs can bind proteins part of the TrxG (68) that antagonize PcG-mediated silencing (149). Interestingly, an antisense lncRNA has been recently involved in recruiting a regulator of DNA demethylation at a specific promoter (150). This process remains still largely unknown and it has only recently been associated to active enzymatic reactions, via TET family of methylcytosine dioxygenases (151, 152). Even in this case, one of the unsolved questions has been how locus-specificity can be achieved. Particularly, DNA demethylation is often restricted to few dinucleotides at the TSS. The precise mechanism, though, through which lncRNAs could direct DNA or chromatin modification has never been described. Indeed in all reported examples, correlations have been described between lncRNA-modifiers associations and loss of modification after lncRNA gene silencing.

Long non-coding regulatory RNAs are supposed to confer binding specificity to modifiers and recruiting them either *in cis* or *in trans*. In the first case, lncRNAs could act directly on sites where they are synthesized without needing to leave DNA. The current hypothesis suggests that the 5′ region of the nascent transcript could bind proteins while the 3′ is transcriptionally lagging, being still tethered to chromatin by RNA polymerase (153). This model is particularly intriguing as through this mechanism, lncRNAs could exert an allele-specific effect, like in the well-studied case of Xist. *In trans* regulation is instead achieved when lncRNAs act modulating genes across great distances or even on different chromosomes (154). Regarding this dichotomy, we must underline once again its artificiality. Indeed, chromosomes fold into complex, three-dimensional territories together with specialized subnuclear bodies. Proteins that are part of the transcriptional or splicing machinery and regulators of these processes group at these foci (155, 156). These structures are not static, but on the contrary, large-scale chromosomal repositioning is observed in response to environmental stimuli or during differentiation (157, 158). Subnuclear movements are of key importance in regulating events like transcription and rearrangement that occur at immunoglobulin loci during B lymphocytes development (159). The dynamic folding of the genome into higher order structure encompasses loci belonging to the same chromosome, even hundreds of kilobases apart, or different ones, bringing together regions that are distant if we consider the genome as linear. Therefore, in this context, it is extremely difficult to discern what regulations are *in cis* or *in trans*, especially when they involve long distance interactions. Intriguingly, lincRNAs have been found to regulate the formation of subnuclear structures, such as NEAT1, required for paraspeckles nucleation (101). LncRNAs can also affect directly the three-dimensional organization of chromosomes enhancing the function of proteins involved in looping formation, like the insulator protein CTCF (160). There are also many examples of lncRNAs involved in three-dimensional local chromatin looping that brings together the ncRNA gene with the region that it regulates within the same chromosome (68, 161). Recently, a lincRNA called Firre has been shown to recruit specific gene loci located on different chromosomes, acting as a docking station for organizing *trans*-chromosomal associations. Consistently, genetic deletion of Firre leads to a loss of proximity of several *trans*-interactions (162). A peculiar type of lncRNA has been described that is transcribed from enhancer regions (eRNAs). Classic enhancer elements therefore likely act through transcription of these lncRNAs that upregulate expression at promoters via the recruitment of Mediator complex (163, 164). Finally, there is increasing evidence that even promoters could be transcribed (165), producing lncRNAs probably involved in the enhancer–promoter loop that was hypothesized years ago but never fully resolved (166).

## Long Non-Coding RNAs in the Adaptive Immune System

The adaptive immune system is an extraordinary context for the study of the role of lincRNAs in differentiation. Indeed, upon antigen stimulation, naïve CD4^+^ T cells differentiate into distinct T-helper subsets that were traditionally considered lineages and defined by a prototypic set of expressed cytokines and master TFs. Recently, this relative simple scenario, although useful, has been subjected to debate. CD4^+^ T cells demonstrated to exhibit substantial plasticity and it has become increasingly clear that they can change the pattern of cytokines and TFs according to the milieu they encounter through their life (167, 168). Not only, in some cases, they can concomitantly express other cytokines and TFs together with their prototypical set. Best examples include IL-10, once thought to specifically identify T_H_2 and now known to be produced also by T_H_1, T_reg_, and T_H_17 cells (169) and IFN-γ, the classic T_H_1 cytokine, frequently released by T_H_17 cells simultaneously with IL-17 (170, 171). Regarding master TFs, T_regs_ can express *Foxp3* (their prototypical TF), but also *ROR*γ*t* (T_H_17 TF) and *Runx3* (172–174); similarly T_FH_ cells can differentiate from FOXP3 positive cells also expressing *Bcl6* (their specific TF) (175, 176). In this context, lncRNAs have a fundamental role in governing flexibility and plasticity or maintenance of cell identity, together with lineage-specific TFs and other ncRNAs. In particular, what is emerging from the literature is that ncRNAs typically act as fine-tuners of fate choices and this seems to be true not only in the immune system. Nonetheless, in the case of CD4^+^ T-cell subsets that are specified but not fully determined, subtle changes in extrinsic signals can reverberate through responsive ncRNAs inducing changes that alter cell phenotype (38, 177, 178). Usually, the stability of lineage identity is achieved through the implementation and inheritance of epigenetic modification, but as mentioned before, lncRNAs can act directly on histone and DNA modifiers redefining this context. Conversely, lncRNAs can also buffer this situation in other conditions, acting as maintainers of cell identity. In the cellular system, lncRNAs can be regarded as minor nodes in a huge interconnected network (179), as they usually interact with few other players. This condition allows them to be more flexible and sensitive to variations without disrupting the whole network integrity (180). This is true both over a very short period, as cells can easily and rapidly adapt to environment, and also over long evolutionary periods, as lncRNAs are among the fastest evolving sequences in the genome (95, 181–183). Conversely, master transcription regulators can be considered highly connected hubs, which confer robustness to the network. Indeed, very few protein-coding genes have been lost from worms to human and mutations are most often pathological (184, 185).

Several single-case or genome-wide studies on lncRNAs in the murine adaptive immune system or cell lines are now available in the literature, whereas only few studies have been conducted until now in the human context. The number of studies that unveiled the function and mechanism of a specific lncRNA is so small that can be counted on one hand (Table 3).

**Table 3:** **Studies on lncRNAs in the adaptive immune system**.

Sample	LncRNAs	Function
Granulocytes, monocytes, NK, B, naïve CD8^+^ and CD4^+^, memory human T cells; *in vitro* polarized precursors T-helper, T_H_0, T_H_1, and T_H_2 human cells	240 lncRNAs associated with autoimmune disease (AID) loci (RNA-seq)	Analysis of the expression profile of the AID-associated lncRNAs (186)
CD4^−^CD8^−^, CD4^+^CD8^+^, CD4^+^CD8^−^, activated CD4^+^ mouse T cells	31423 lncRNAs (lncRNA microarray)	Expression analysis and prediction of function (187)
17 T-cell leukemia cell lines	Thy-ncR1 (expression profiling of 10 thymus-specific ncRNA)	Enriched in human immature cells; acts as a cytoplasmic riboregulator that reduces the level of MFAP4 mRNA (188)
Naïve, memory, activated, non-activated mouse CD8^+^ T cells	Over 1000 mouse and human lncRNAs (microarray)	Expression and conservation analysis (37)
CD4^−^CD8^−^, CD4^+^CD8^+^, CD4^+^, CD8^+^ mouse thymic T cells, and thymus-derived T_reg_ cells. *In vitro* differentiated T_H_1, T_H_2, T_H_17, and induced T_reg_	1524 lincRNA genes (RNA-seq); LincR-Ccr2-5′AS	Expression analysis and ChIP-seq data analysis to identify lincRNA genes and possible regulators. LincR-Ccr2-5′AS is T_H_2-specific and it reduces the expression of *Ccr1*, *Ccr2*, *Ccr3*, and *Ccr5*. It contributes to the migration of T_H_2 cells (189)
Infected Namalwa B lymphocytes	IFNA1-AS	Cytoplasmic post-transcriptional stabilization of IFN-α1 RNA masking a miRNA-binding site (190)
Jurkat cells, primary lymphomas, lymphoma cell lines, CD19^+^ B cells	Saf/FAS-AS1	Regulates the alternative splicing of Fas which is impaired in non-Hodgkin’s lymphomas associated with poor prognosis (191, 192)
Activated human CD4^+^ T cells	*BIC* RNA (EST library analysis)	Proto-oncogene, induced upon activation, sensitive to immunosuppressive drugs (193)
Jurkat cells	NRON (shRNA knock-down screening)	Regulates NFAT subcellular localization as part of an RNA–protein complex (84)
CEM-C7 CKM1, jurkat JKM1, human primary lymphocytes	GAS5	Necessary and sufficient for growth arrest. Acts competing from GREs (71, 106)
Human CD4^+^, CD8^+^ cells, PBMC	*NTT*	Unknown, it shows a similar expression pattern to *IFN*γ*R* (194)
Thymocytes	TEA	Instruct the activity of Jα promoters and recombination (103, 195)
Human T_H_1 cells	NeST/Tmevpg1/IFNG-AS1	Dependent on STAT4, T-bet, and NFκB. Contributes to *Ifng* expression by binding WDR5 and alter H3K4me3 (196, 197)
Human primary CD4^+^ and CD8^+^ T cells, primary and polarized (from CD4^+^ and CD8^+^ T) CD4^+^ CM, T_H_1, T_H_2, T_H_17, and T_reg_ cells; neutrophils, basophils, CD8^+^ CM, B cells	*GATA3-AS1*	Specifically expressed in T_H_ 2 cells (198)

The importance of the studies in the human immune system is underlined by the fact that the differences between experimental animal models and human are still subject of debate in terms of immunologic responses (199–201). Moreover, there are increasing evidences that ncRNAs are poorly conserved between animal models and human (202, 203). In particular, lncRNAs are really fast-evolving elements as demonstrated by the fact that over 80% of the human lncRNAs that arose in the primate lineage, only 3% are conserved across tetrapods and most mammalian lncRNAs lack known orthologs outside vertebrates (97). In detail, even between mouse and human, lncRNAs are poorly conserved (204–206). Despite their rapid evolution, lncRNAs are selected more than neutral sequences and in particular more than intergenic regions, but significantly less than mRNAs (96, 97, 207). It must be underlined that the conservation rate reported could be overestimated: substitution rates are derived from whole-genome alignment and based on the assumption that even segment of homologies imply that that segment belongs to the same RNA class, but this is not necessarily the case. Indeed, it could be that in another genome context a specific lncRNA gene segment is transcribed and processed as part of a protein-coding RNA (208). A striking example is Hotair that is involved in the regulation of the highly conserved cluster of *Hox* genes (68). The human lincRNA is conserved in the mouse genome (209), nonetheless only the 3′ region is effectively part of the murine homolog (183). The importance of studying lincRNAs specifically within the human immune system derives from these considerations, but this field is still poorly investigated. The majority of the studies focused on the innate immune system (210–212) or analyzed pathological situations, such as cancer-related lncRNAs (192, 213) or responses to specific infections (102, 214–216), mostly in mice. The first functional study that focused on the adaptive immune system, and in particular on T_H_1 and T_H_2 lymphocytes, involved a lincRNA, Tmevpg1, that is selectively expressed in T_H_1 cells via STAT4 and T-bet, both in mouse and human. It participates in the induction of IFN-γ expression strictly in response to T_H_1 differentiation program and not in other cellular contexts. These results highlight once again the complexity of gene expression regulatory network and the specificity of action of lincRNAs (196). Another paper described a lincRNA, GATA3-AS1, specifically expressed in primary T_H_2 cells and hypothesized its co-regulation with GATA3 (198). GAS5, expressed in human T lymphocytes, is degraded in optimal growth conditions, but it accumulates contributing to growth arrest in starving conditions (107). In this situation, it competes with GRs DNA-binding sequences, suppressing GR-mediated transcription (106). Broader studies have been performed on the CD8^+^ T cell transcriptome (37), and recently on CD4^+^ T lymphocytes (189), but still on mice models. In B cells, chromatin remodeling associated with V(D)J recombination has been potentially linked to a widespread antisense intergenic transcription that occurs in the variable (V) region of the immunoglobulin heavy chain (Igh) locus (104, 105). So far, no studies have been published that performed a deep transcriptomic analysis on human primary lymphocytes from healthy donors, identifying lncRNAs fundamental for differentiation processes. These few examples are just clues of the importance that lincRNA could have for the proper function also of the human immune system and prompt to a deeper analysis of their role in this particularly intriguing context.

## Long Non-Coding RNAs as Epigenetic Modulators in Lymphocyte Differentiation

Traditionally, the secretion of IFN-γ and TNF-α characterizes T_H_1 lymphocytes, whereas IL-4, IL-5, and IL-13 are considered prototypic cytokines secreted by T_H_2 cells. According to this classic paradigm, these differences underline the different functions exerted by these lymphocytes: T_H_1 are considered as important to eliminate intracellular bacteria and viruses, whereas T_H_2 to resist parasitic infections (217). The advantage of solid *in vitro* differentiation protocols allowed a deep understanding of the genetic mechanisms governing these cells. Since the discovery of this dichotomy, other cell subsets have been identified, but this T_H_1/T_H_2 paradigm was undoubtedly useful. Therefore, it is not a case that among the few lncRNAs identified in the immune system, many of those functionally characterized have been described in these two cell subsets. Nevertheless as mentioned before, just one lincRNA, Tmevpg1 (also known as NeST or IFNG-AS1) has been characterized in deep. Tmevpg1 is located proximal to IFN-γ gene both in mice and humans, antisense and convergently transcribed respect to the neighboring gene and plays a role in chromatin remodeling. This transcript is a T_H_1-specific lincRNA: it requires STAT4 and T-bet for being transcribed and is also bound by CTCF and cohesin during lineage-specific induction (196). Therefore, Tmevpg1 is directly dependent on the activation of a T_H_1-polarizing transcriptional program, in which the presence of IL-12 leads to the activation of the JAK/STAT pathway via STAT4 (and STAT1) that induces the expression of T-bet. Interestingly, *Tmevpg1* gene harbor sequences regulated by histone acetylation and DNase I hypersensitive sites found in T_H_1 but not T_H_2 cells (218, 219). Tmevpg1, in its turn, plays a direct part in defining the proper T_H_1 cytokine expression pattern, influencing *Ifng* transcription in the presence of T-bet (196), via H3K4 trimethylation by WDR5 binding in mice models (197).

Given the increasing number of lncRNAs described in different cellular contexts and the high number of specific lincRNAs expressed in the different lymphocytes subsets identified with the aforementioned RNA-seq analysis, many more lincRNAs will likely be characterized in the future with a relevant function in the human immune system. A major limitation, though, in the studies on lncRNAs is that there is little biological knowledge on the biochemical or molecular function of lncRNA genes. Compared to classical protein-coding gene studies, hints on their functions cannot be gained simply by the analysis of their primary sequence and application of computational methods to infer lncRNA function are also still in their infancy. As lincRNAs have been reported to influence the expression of neighboring genes (25, 26, 28, 39), one possible approach to investigate their putative function is to focus on lymphocyte lincRNAs proximal to protein-coding genes involved in key cell-functions.

**Figure 2:**
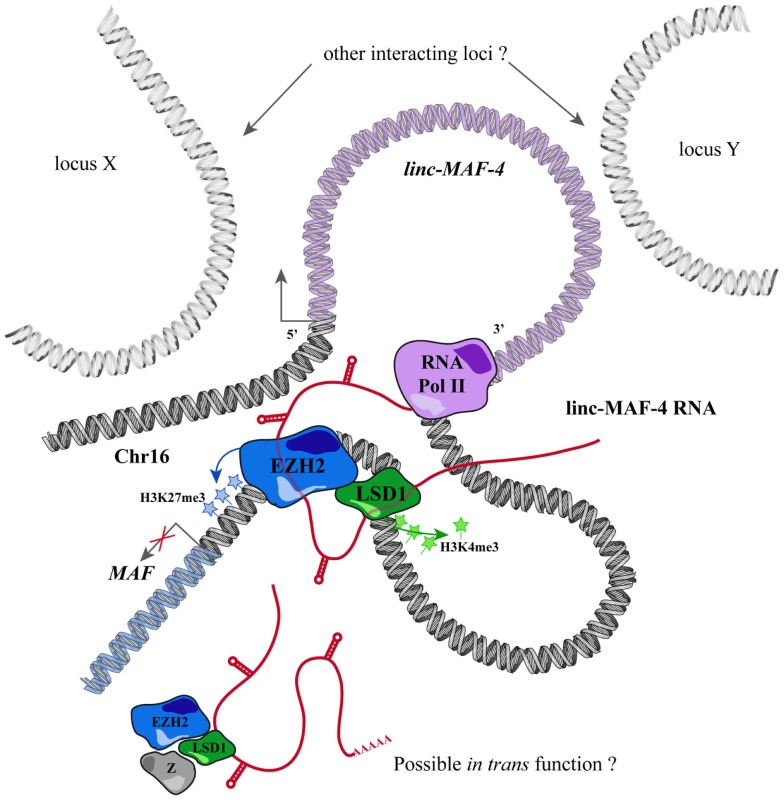
**Mechanism of action proposed for linc-MAF-4**.

Through this approach, we identified a T_H_1-specific lincRNA that localized ~140 kb upstream to *MAF* that was therefore called linc-MAF-4. MAF is a TF involved in T_H_2 differentiation and required for the efficient secretion of IL-4 by T_H_17 and the proper development of T_FH_ cells (220–222). Intriguingly, the expression of *linc-MAF-4* is negatively correlated with respect to the expression of *MAF*: linc-MAF-4 expression is high and specific in T_H_1 lymphocytes, where *MAF* is lowly expressed whereas in T_H_2 cells the expression of *linc-MAF-4* is extremely low and *MAF* is highly expressed. Coherently, linc-MAF-4 knock-down in naïve CD4^+^ T cells increased the expression of *MAF* and interestingly induced a more general skewing of the whole transcriptomic profile of these cells toward a T_H_2-like fate (63). The regulation exerted by linc-MAF-4 on *MAF* gene was analyzed in more detail and this lincRNA proved to modulate *MAF* expression *in cis*, as hypothesized by expression analysis. Linc-MAF-4 exerts this regulation by exploiting a chromatin loop that brings its genomic region close to the promoter of *MAF* gene. Indeed, the chromatin organization of this region allows linc-MAF-4 transcript to recruit chromatin remodelers that inhibit *MAF* transcription. In particular, linc-MAF-4 was found to associate with EZH2, key enzymatic subunit of the PRC2 complex, and LSD1. These proteins methylate H3K27 and demethylate H3K4, respectively: two histones modifications that code for transcriptional repression. (63). A similar mechanism was described for other lincRNAs, such as HOTAIR and MEG3 (154, 223) but never before for other lncRNAs expressed in the adaptive immune system (Figure 2).

Changes of lincRNAs expression during naïve to memory CD8^+^ T-cell differentiation (37) and during naïve CD4^+^ T cells differentiation into distinct helper T-cell lineages (189) have been described in the mouse immune system. linc-MAF-4, though, is, to our knowledge, the first example of a lincRNA playing a role in the proper differentiation of human T_H_1 cells, suggesting that, besides cytokines and TFs, lncRNAs take part in the T_H_1 differentiation program as already shown in many other cell types. At this point, an obvious question arises: to what extent are these cells plastic? These findings are evidences that it is possible to re-direct the differentiation path of naïve CD4^+^ T cells acting on their lincRNA content. Nevertheless, could it be possible to modulate already committed cells? We would expect that the mere down-regulation of a lincRNA would not be sufficient nor a lincRNA knock-out: as mentioned before, lincRNAs are minor nodes in a huge interconnected network composed by feedback mechanisms and epigenetic marks that act stabilizing a pre-existent differentiation status. However, a modulation in lincRNA content may be sufficient to make these cells more responsive to environmental cues that could overcome stabilizing forces, inducing a sort of trans-differentiation event. Functional characterization of other lncRNAs is required to address this crucial issue and to assess the extent of their contribution to cell differentiation and to the maintenance of cell identity in human lymphocytes. Based on what we discussed so far on lncRNA functions and cell-specificity, we believe that future studies will show how these molecules could be capitalized as new molecular targets for the development of novel and highly specific therapies for diseases, such as autoimmunity, immunodeficiencies, allergy, and cancer.

## Conflict of Interest Statement

The authors declare that the research was conducted in the absence of any commercial or financial relationships that could be construed as a potential conflict of interest.

## References

[1] Derrien T Johnson R Bussotti G Tanzer A Djebali S Tilgner H The GENCODE v7 catalog of human long noncoding RNAs: analysis of their gene structure, evolution, and expression. Genome Res (2012) 22(9):1775–89. 10.1101/gr.132159.111 22955988 PMC3431493

[2] Taft RJ Pheasant M Mattick JS. The relationship between non-protein-coding DNA and eukaryotic complexity. Bioessays (2007) 29(3):288–99. 10.1002/bies.20544 17295292

[3] Hoagland MB Keller EB Zamecnik PC Enzymatic carboxyl activation of amino acids. J Biol Chem (1956) 218(1):345–58. 13278342

[4] Pasquinelli AE Reinhart BJ Slack F Martindale MQ Kuroda MI Maller B Conservation of the sequence and temporal expression of let-7 heterochronic regulatory RNA. Nature (2000) 408(6808):86–9. 10.1038/35040556 11081512

[5] Baumjohann D Ansel KM. MicroRNA-mediated regulation of T helper cell differentiation and plasticity. Nat Rev Immunol (2013) 13(9):666–78. 10.1038/nri3494 23907446 PMC3980848

[6] Bronevetsky Y Ansel KM. Regulation of miRNA biogenesis and turnover in the immune system. Immunol Rev (2013) 253(1):304–16. 10.1111/imr.12059 23550654 PMC3621012

[7] Monticelli S. MicroRNAs in T helper cell differentiation and plasticity. Semin Immunol (2013) 25(4):291–8. 10.1016/j.smim.2013.10.015 24216176

[8] Bratkovič T Rogelj B. The many faces of small nucleolar RNAs. Biochim Biophys Acta (2014) 1839(6):438–43. 10.1016/j.bbagrm.2014.04.009 24735946

[9] Dieci G Preti M Montanini B. Eukaryotic snoRNAs: a paradigm for gene expression flexibility. Genomics (2009) 94(2):83–8. 10.1016/j.ygeno.2009.05.002 19446021

[10] Lui L Lowe T. Small nucleolar RNAs and RNA-guided post-transcriptional modification. Essays Biochem (2013) 54:53–77. 10.1042/bse0540053 23829527

[11] Valadkhan S Gunawardane LS Role of small nuclear RNAs in eukaryotic gene expression. Essays Biochem (2013) 54:79–90 10.1042/bse0540079 23829528 PMC11246792

[12] Bamezai S Rawat VPS Buske C. Concise review: the Piwi-piRNA axis: pivotal beyond transposon silencing. Stem Cells (2012) 30(12):2603–11. 10.1002/stem.1237 22996918

[13] Ku H-Y Lin H. PIWI proteins and their interactors in piRNA biogenesis, germline development and gene expression. Natl Sci Rev (2014) 1(2):205–18. 10.1093/nsr/nwu014 25512877 PMC4265212

[14] Luteijn MJ Ketting RF. PIWI-interacting RNAs: from generation to transgenerational epigenetics. Nat Rev Genet (2013) 14(8):523–34. 10.1038/nrg3495 23797853

[15] Kapranov P Cheng J Dike S Nix DA Duttagupta R Willingham AT RNA maps reveal new RNA classes and a possible function for pervasive transcription. Science (2007) 316(5830):1484–8. 10.1126/science.1138341 17510325

[16] Taft RJ Kaplan CD Simons C Mattick JS. Evolution, biogenesis and function of promoter-associated RNAs. Cell Cycle (2009) 8(15):2332–8. 10.4161/cc.8.15.9154 19597344

[17] Carthew RW Sontheimer EJ. Origins and mechanisms of miRNAs and siRNAs. Cell (2009) 136(4):642–55. 10.1016/j.cell.2009.01.035 19239886 PMC2675692

[18] Svoboda P. Renaissance of mammalian endogenous RNAi. FEBS Lett (2014) 588(15):2550–6. 10.1016/j.febslet.2014.05.030 24873877

[19] Taft RJ Glazov EA Cloonan N Simons C Stephen S Faulkner GJ Tiny RNAs associated with transcription start sites in animals. Nat Genet (2009) 41(5):572–8. 10.1038/ng.312 19377478

[20] Taft RJ Simons C Nahkuri S Oey H Korbie DJ Mercer TR Nuclear-localized tiny RNAs are associated with transcription initiation and splice sites in metazoans. Nat Struct Mol Biol (2010) 17(8):1030–4. 10.1038/nsmb.1841 20622877

[21] Djebali S Davis CA Merkel A Dobin A Lassmann T Mortazavi AM Landscape of transcription in human cells. Nature (2012) 489(7414):101–8 10.1038/nature11233 22955620 PMC3684276

[22] Faghihi MA Wahlestedt C. Regulatory roles of natural antisense transcripts. Nat Rev Mol Cell Biol (2009) 10(9):637–43. 10.1038/nrm2738 19638999 PMC2850559

[23] Kurokawa R. Promoter-associated long noncoding RNAs repress transcription through a RNA binding protein TLS. Adv Exp Med Biol (2011) 722:196–208. 10.1007/978-1-4614-0332-6_12 21915790

[24] Zaphiropoulos PG A promoter-associated RNA downregulates the oncogenic GLI1 transcription factor in rhabdomyosarcoma cells. RNA Dis (2014) 1(1):e254 10.14800/rd.254

[25] Preker P Nielsen J Kammler S Lykke-Andersen S Christensen MS Mapendano CK RNA exosome depletion reveals transcription upstream of active human promoters. Science (2008) 322(5909):1851–4. 10.1126/science.1164096 19056938

[26] Imamura T Yamamoto S Ohgane J Hattori N Tanaka S Shiota K. Non-coding RNA directed DNA demethylation of Sphk1 CpG island. Biochem Biophys Res Commun (2004) 322(2):593–600. 10.1016/j.bbrc.2004.07.159 15325271

[27] Preker P Almvig K Christensen MS Valen E Mapendano CK Sandelin A PROMoter uPstream Transcripts share characteristics with mRNAs and are produced upstream of all three major types of mammalian promoters. Nucleic Acids Res (2011) 39(16):7179–93. 10.1093/nar/gkr370 21596787 PMC3167610

[28] Peng JC Shen J Ran ZH. Transcribed ultraconserved region in human cancers. RNA Biol (2013) 10(12):1771–7. 10.4161/rna.26995 24384562 PMC3917980

[29] Louro R Smirnova AS Verjovski-Almeida S. Long intronic noncoding RNA transcription: expression noise or expression choice? Genomics (2009) 93(4):291–8. 10.1016/j.ygeno.2008.11.009 19071207

[30] Lam MT Li W Rosenfeld MG Glass CK. Enhancer RNAs and regulated transcriptional programs. Trends Biochem Sci (2014) 39(4):170–82. 10.1016/j.tibs.2014.02.007 24674738 PMC4266492

[31] Koch F Fenouil R Gut M Cauchy P Albert TK Zacarias-Cabeza J Transcription initiation platforms and GTF recruitment at tissue-specific enhancers and promoters. Nat Struct Mol Biol (2011) 18(8):956–63. 10.1038/nsmb.2085 21765417

[32] Natoli G Andrau JC. Noncoding transcription at enhancers: general principles and functional models. Annu Rev Genet (2012) 46:1–19. 10.1146/annurev-genet-110711-155459 22905871

[33] Rinn JL Chang HY. Genome regulation by long noncoding RNAs. Annu Rev Biochem (2012) 81:145–66. 10.1146/annurev-biochem-051410-92902 22663078 PMC3858397

[34] Merry C Niland C Khalil A Diverse functions and mechanisms of mammalian long noncoding RNAs. In: Carmichael GG, editor. Regulatory Non-Coding RNAs: Methods in Molecular Biology. Vol. 1206 New York, NY: Springer (2015). p. 1–14. 10.1007/978-1-4939-1369-5_1 25240882

[35] Mercer TR Wilhelm D Dinger ME Soldà G Korbie DJ Glazov EA Expression of distinct RNAs from 3’ untranslated regions. Nucleic Acids Res (2011) 39(6):2393–403. 10.1093/nar/gkq1158 21075793 PMC3064787

[36] Lasda E Parker R. Circular RNAs: diversity of form and function. RNA (2014) 20(12):1829–42. 10.1261/rna.047126.114 25404635 PMC4238349

[37] Pang KC Dinger ME Mercer TR Malquori L Grimmond SM Chen W Genome-wide identification of long noncoding RNAs in CD8+ T cells. J Immunol (2009) 182(12):7738–48. 10.4049/jimmunol.0900603 19494298

[38] Pagani M Rossetti G Panzeri I de Candia P Bonnal RJ Rossi RL Role of microRNAs and long-non-coding RNAs in CD4(+) T-cell differentiation. Immunol Rev (2013) 253(1):82–96. 10.1111/imr.12055 23550640

[39] Guttman M Amit I Garber M French C Lin MF Feldser D Chromatin signature reveals over a thousand highly conserved large non-coding RNAs in mammals. Nature (2009) 458(7235):223–7. 10.1038/nature07672 19182780 PMC2754849

[40] Dieci G Conti A Pagano A Carnevali D. Identification of RNA polymerase III-transcribed genes in eukaryotic genomes. Biochim Biophys Acta (2013) 1829(3–4):296–305. 10.1016/j.bbagrm.2012.09.010 23041497

[41] Lin R Maeda S Liu C Karin M Edgington TS. A large noncoding RNA is a marker for murine hepatocellular carcinomas and a spectrum of human carcinomas. Oncogene (2007) 26(6):851–8. 10.1038/sj.onc.1209846 16878148

[42] Galindo MI Pueyo JI Fouix S Bishop SA Couso JP. Peptides encoded by short ORFs control development and define a new eukaryotic gene family. PLoS Biol (2007) 5(5):e106. 10.1371/journal.pbio.0050106 17439302 PMC1852585

[43] Lanz RB McKenna NJ Onate SA Albrecht U Wong J Tsai SY A steroid receptor coactivator, SRA, functions as an RNA and is present in an SRC-1 complex. Cell (1999) 97(1):17–27. 10.1016/S0092-8674(00)80711-4 10199399

[44] Ingolia NT Brar GA Stern-Ginossar N Harris MS Talhouarne GJ Jackson SE Ribosome profiling reveals pervasive translation outside of annotated protein-coding genes. Cell Rep (2014) 8(5):1365–79. 10.1016/j.celrep.2014.07.045 25159147 PMC4216110

[45] Banfai B Jia H Khatun J Wood E Risk B Gundling WE Jr Long noncoding RNAs are rarely translated in two human cell lines. Genome Res (2012) 22(9):1646–57. 10.1101/gr.134767.111 22955977 PMC3431482

[46] Lee S Liu B Lee S Huang SX Shen B Qian SB. Global mapping of translation initiation sites in mammalian cells at single-nucleotide resolution. Proc Natl Acad Sci U S A (2012) 109(37):E2424–32. 10.1073/pnas.1207846109 22927429 PMC3443142

[47] Slavoff SA Mitchell AJ Schwaid AG Cabili MN Ma J Levin JZ Peptidomic discovery of short open reading frame-encoded peptides in human cells. Nat Chem Biol (2013) 9(1):59–64. 10.1038/nchembio.1120 23160002 PMC3625679

[48] Andrews SJ Rothnagel JA. Emerging evidence for functional peptides encoded by short open reading frames. Nat Rev Genet (2014) 15(3):193–204. 10.1038/nrg3520 24514441

[49] Ulveling D Francastel C Hube F. When one is better than two: RNA with dual functions. Biochimie (2011) 93(4):633–44. 10.1016/j.biochi.2010.11.004 21111023

[50] Poliseno L Salmena L Zhang J Carver B Haveman WJ Pandolfi PP. A coding-independent function of gene and pseudogene mRNAs regulates tumour biology. Nature (2010) 465(7301):1033–8. 10.1038/nature09144 20577206 PMC3206313

[51] Duret L Chureau C Samain S Weissenbach J Avner P. The Xist RNA gene evolved in eutherians by pseudogenization of a protein-coding gene. Science (2006) 312(5780):1653–5. 10.1126/science.1126316 16778056

[52] Elisaphenko EA Kolesnikov NN Shevchenko AI Rogozin IB Nesterova TB Brockdorff N A dual origin of the *Xist* gene from a protein-coding gene and a set of transposable elements. PLoS One (2008) 3(6):e2521. 10.1371/journal.pone.0002521 18575625 PMC2430539

[53] Horvath JE Sheedy CB Merrett SL Diallo AB Swofford DL Program NCS Comparative analysis of the primate X-inactivation center region and reconstruction of the ancestral primate XIST locus. Genome Res (2011) 21(6):850–62. 10.1101/gr.111849.110 21518738 PMC3106318

[54] Romito A Rougeulle C. Origin and evolution of the long non-coding genes in the X-inactivation center. Biochimie (2011) 93(11):1935–42. 10.1016/j.biochi.2011.07.009 21820484

[55] Bussotti G Notredame C Enright A. Detecting and comparing non-coding RNAs in the high-throughput era. Int J Mol Sci (2013) 14(8):15423–58. 10.3390/ijms140815423 23887659 PMC3759867

[56] Nigro JM Cho KR Fearon ER Kern SE Ruppert JM Oliner JD Scrambled exons. Cell (1991) 64(3):607–13. 10.1016/0092-8674(91)90244-S 1991322

[57] Beltran M Puig I Peña C García JM Álvarez AB Peña R A natural antisense transcript regulates Zeb2/Sip1 gene expression during Snail1-induced epithelial-mesenchymal transition. Genes Dev (2008) 22(6):756–69. 10.1101/gad.455708 18347095 PMC2275429

[58] Martick M Horan LH Noller HF Scott WG. A discontinuous hammerhead ribozyme embedded in a mammalian messenger RNA. Nature (2008) 454(7206):899–902. 10.1038/nature07117 18615019 PMC2612532

[59] Mercer TR Gerhardt DJ Dinger ME Crawford J Trapnell C Jeddeloh JA Targeted RNA sequencing reveals the deep complexity of the human transcriptome. Nat Biotech (2012) 30(1):99–104. 10.1038/nbt.2024 22081020 PMC3710462

[60] Katayama S Tomaru Y Kasukawa T Waki K Nakanishi M Nakamura M Antisense transcription in the mammalian transcriptome. Science (2005) 309(5740):1564–6 10.1126/science.1112009 16141073

[61] Li Z Rana TM. Decoding the noncoding: prospective of lncRNA-mediated innate immune regulation. RNA Biol (2014) 11(8):979–85. 10.4161/rna.29937 25482890 PMC4615744

[62] Imamura K Akimitsu N. Long non-coding RNAs involved in immune responses. Front Immunol (2014) 5:573. 10.3389/fimmu.2014.00573 25431574 PMC4230175

[63] Ranzani VRG Panzeri I Arrigoni A Bonnal R Curti S Gruarin P LincRNA landscape in human lymphocytes highlights regulation of T cell differentiation by linc-MAF-4. Nat Immunol (2015) 16(3):318–25. 10.1038/ni.3093 25621826 PMC4333215

[64] Kapusta A Kronenberg Z Lynch VJ Zhuo X Ramsay L Bourque G Transposable elements are major contributors to the origin, diversification, and regulation of vertebrate long noncoding RNAs. PLoS Genet (2013) 9(4):e1003470. 10.1371/journal.pgen.1003470 23637635 PMC3636048

[65] Kelley D Rinn J. Transposable elements reveal a stem cell-specific class of long noncoding RNAs. Genome Biol (2012) 13(11):R107. 10.1186/gb-2012-13-11-r107 23181609 PMC3580499

[66] Lin N Chang KY Li Z Gates K Rana ZA Dang J An evolutionarily conserved long noncoding RNA TUNA controls pluripotency and neural lineage commitment. Mol Cell (2014) 53(6):1005–19. 10.1016/j.molcel.2014.01.021 24530304 PMC4010157

[67] Klattenhoff CA Scheuermann JC Surface LE Bradley RK Fields PA Steinhauser ML Braveheart, a long noncoding RNA required for cardiovascular lineage commitment. Cell (2013) 152(3):570–83. 10.1016/j.cell.2013.01.003 23352431 PMC3563769

[68] Wang KC Yang YW Liu B Sanyal A Corces-Zimmerman R Chen Y A long noncoding RNA maintains active chromatin to coordinate homeotic gene expression. Nature (2011) 472(7341):120–4. 10.1038/nature09819 21423168 PMC3670758

[69] Keniry A Oxley D Monnier P Kyba M Dandolo L Smits G The H19 lincRNA is a developmental reservoir of miR-675 that suppresses growth and Igf1r. Nat Cell Biol (2012) 14(7):659–65. 10.1038/ncb2521 22684254 PMC3389517

[70] Venkatraman A He XC Thorvaldsen JL Sugimura R Perry JM Tao F Maternal imprinting at the H19-Igf2 locus maintains adult haematopoietic stem cell quiescence. Nature (2013) 500(7462):345–9. 10.1038/nature12303 23863936 PMC3896866

[71] Mourtada-Maarabouni M Hedge VL Kirkham L Farzaneh F Williams GT. Growth arrest in human T-cells is controlled by the non-coding RNA growth-arrest-specific transcript 5 (GAS5). J Cell Sci (2008) 121(7):939–46. 10.1242/jcs.024646 18354083

[72] Pickard MR Mourtada-Maarabouni M Williams GT. Long non-coding RNA GAS5 regulates apoptosis in prostate cancer cell lines. Biochim Biophys Acta (2013) 1832(10):1613–23. 10.1016/j.bbadis.2013.05.005 23676682

[73] Rossi MN Antonangeli F. LncRNAs: new players in apoptosis control. Int J Cell Biol (2014) 2014:7. 10.1155/2014/473857 24627686 PMC3929073

[74] Yin D He X Zhang E Kong R De W Zhang Z. Long noncoding RNA GAS5 affects cell proliferation and predicts a poor prognosis in patients with colorectal cancer. Med Oncol (2014) 31(11):253. 10.1007/s12032-014-0253-8 25326054

[75] Cesana M Cacchiarelli D Legnini I Santini T Sthandier O Chinappi M A long noncoding RNA controls muscle differentiation by functioning as a competing endogenous RNA. Cell (2011) 147(2):358–69. 10.1016/j.cell.2011.09.028 22000014 PMC3234495

[76] Fatica A Bozzoni I. Long non-coding RNAs: new players in cell differentiation and development. Nat Rev Genet (2014) 15(1):7–21. 10.1038/nrg3606 24296535

[77] Hu W Alvarez-Dominguez JR Lodish HF Regulation of mammalian cell differentiation by long non-coding RNAs. EMBO Rep (2012) 13(11):971–83 10.1038/embor.2012.145 23070366 PMC3492712

[78] Lee Jeannie T Bartolomei Marisa S. X-inactivation, imprinting, and long noncoding RNAs in health and disease. Cell (2013) 152(6):1308–23. 10.1016/j.cell.2013.02.016 23498939

[79] Mitsuya K Meguro M Lee MP Katoh M Schulz TC Kugoh H LIT1, an imprinted antisense RNA in the human KvLQT1 locus identified by screening for differentially expressed transcripts using monochromosomal hybrids. Hum Mol Genet (1999) 8(7):1209–17. 10.1093/hmg/8.7.1209 10369866

[80] Zhang H Zeitz MJ Wang H Niu B Ge S Li W Long noncoding RNA-mediated intrachromosomal interactions promote imprinting at the Kcnq1 locus. J Cell Biol (2014) 204(1):61–75. 10.1083/jcb.201304152 24395636 PMC3882787

[81] Gendrel AV Heard E. Noncoding RNAs and epigenetic mechanisms during X-chromosome inactivation. Annu Rev Cell Dev Biol (2014) 30:561–80. 10.1146/annurev-cellbio-101512-122415 25000994

[82] Khalil AM Guttman M Huarte M Garber M Raj A Rivea Morales D Many human large intergenic noncoding RNAs associate with chromatin-modifying complexes and affect gene expression. Proc Natl Acad Sci U S A (2009) 106(28):11667–72. 10.1073/pnas.0904715106 19571010 PMC2704857

[83] Salmena L Poliseno L Tay Y Kats L Pandolfi Pier P. A ceRNA hypothesis: the Rosetta stone of a hidden RNA language? Cell (2011) 146(3):353–8. 10.1016/j.cell.2011.07.014 21802130 PMC3235919

[84] Willingham AT Orth AP Batalov S Peters EC Wen BG Aza-Blanc P A strategy for probing the function of noncoding RNAs finds a repressor of NFAT. Science (2005) 309(5740):1570–3. 10.1126/science.1115901 16141075

[85] Carrieri C Cimatti L Biagioli M Beugnet A Zucchelli S Fedele S Long non-coding antisense RNA controls Uchl1 translation through an embedded SINEB2 repeat. Nature (2012) 491(7424):454–7. 10.1038/nature11508 23064229

[86] Tripathi V Ellis JD Shen Z Song DY Pan Q Watt AT The nuclear-retained noncoding RNA MALAT1 regulates alternative splicing by modulating SR splicing factor phosphorylation. Mol Cell (2010) 39(6):925–38. 10.1016/j.molcel.2010.08.011 20797886 PMC4158944

[87] Conrad NK. The emerging role of triple helices in RNA biology. Wiley Interdiscip Rev RNA (2014) 5(1):15–29. 10.1002/wrna.1194 24115594 PMC4721660

[88] Cruz JA Westhof E The dynamic landscapes of RNA architecture. Cell (2009) 136(4):604–9 10.1016/j.cell.2009.02.003 19239882

[89] Lescoute A Westhof E. Topology of three-way junctions in folded RNAs. RNA (2006) 12(1):83–93. 10.1261/rna.2208106 16373494 PMC1370888

[90] Jia G Fu Y Zhao X Dai Q Zheng G Yang Y N6-Methyladenosine in nuclear RNA is a major substrate of the obesity-associated FTO. Nat Chem Biol (2011) 7(12):885–7. 10.1038/nchembio.687 22002720 PMC3218240

[91] Yang Y Zhou X Jin Y. ADAR-mediated RNA editing in non-coding RNA sequences. Sci China Life Sci (2013) 56(10):944–52. 10.1007/s11427-013-4546-5 24008387

[92] Squires JE Patel HR Nousch M Sibbritt T Humphreys DT Parker BJ Widespread occurrence of 5-methylcytosine in human coding and non-coding RNA. Nucleic Acids Res (2012) 40(11):5023–33. 10.1093/nar/gks144 22344696 PMC3367185

[93] Chi SW Hannon GJ Darnell RB. An alternative mode of microRNA target recognition. Nat Struct Mol Biol (2012) 19(3):321–7. 10.1038/nsmb.2230 22343717 PMC3541676

[94] Mariner PD Walters RD Espinoza CA Drullinger LF Wagner SD Kugel JF Human Alu RNA is a modular transacting repressor of mRNA transcription during heat shock. Mol Cell (2008) 29(4):499–509. 10.1016/j.molcel.2007.12.013 18313387

[95] Kutter C Watt S Stefflova K Wilson MD Goncalves A Ponting CP Rapid turnover of long noncoding RNAs and the evolution of gene expression. PLoS Genet (2012) 8(7):e1002841. 10.1371/journal.pgen.1002841 22844254 PMC3406015

[96] Ponjavic J Ponting CP Lunter G. Functionality or transcriptional noise? Evidence for selection within long noncoding RNAs. Genome Res (2007) 17(5):556–65. 10.1101/gr.6036807 17387145 PMC1855172

[97] Marques AC Ponting CP. Intergenic lncRNAs and the evolution of gene expression. Curr Opin Genet Dev (2014) 27(0):48–53. 10.1016/j.gde.2014.03.009 24852186

[98] Engreitz Jesse M Sirokman K McDonel P Shishkin AA Surka C Russell P RNA-RNA interactions enable specific targeting of noncoding RNAs to nascent pre-mRNAs and chromatin sites. Cell (2014) 159(1):188–99. 10.1016/j.cell.2014.08.018 25259926 PMC4177037

[99] Chu C Qu K Zhong FL Artandi SE Chang HY. Genomic maps of long noncoding RNA occupancy reveal principles of RNA-chromatin interactions. Mol Cell (2011) 44(4):667–78. 10.1016/j.molcel.2011.08.027 21963238 PMC3249421

[100] Schmitz KM Mayer C Postepska A Grummt I. Interaction of noncoding RNA with the rDNA promoter mediates recruitment of DNMT3b and silencing of rRNA genes. Genes Dev (2010) 24(20):2264–9. 10.1101/gad.590910 20952535 PMC2956204

[101] Clemson CM Hutchinson JN Sara SA Ensminger AW Fox AH Chess A An architectural role for a nuclear noncoding RNA: NEAT1 RNA is essential for the structure of paraspeckles. Mol Cell (2009) 33(6):717–26. 10.1016/j.molcel.2009.01.026 19217333 PMC2696186

[102] Imamura K Imamachi N Akizuki G Kumakura M Kawaguchi A Nagata K Long noncoding RNA NEAT1-dependent SFPQ relocation from promoter region to paraspeckle mediates IL8 expression upon immune stimuli. Mol Cell (2014) 53(3):393–406. 10.1016/j.molcel.2014.01.009 24507715

[103] Abarrategui I Krangel MS. Noncoding transcription controls downstream promoters to regulate T-cell receptor alpha recombination. EMBO J (2007) 26(20):4380–90. 10.1038/sj.emboj.7601866 17882258 PMC2034674

[104] Bolland DJ Wood AL Johnston CM Bunting SF Morgan G Chakalova L Antisense intergenic transcription in V(D)J recombination. Nat Immunol (2004) 5(6):630–7. 10.1038/ni1068 15107847

[105] Verma-Gaur J Torkamani A Schaffer L Head SR Schork NJ Feeney AJ. Noncoding transcription within the Igh distal V(H) region at PAIR elements affects the 3D structure of the Igh locus in pro-B cells. Proc Natl Acad Sci U S A (2012) 109(42):17004–9. 10.1073/pnas.1208398109 23027941 PMC3479473

[106] Kino T Hurt DE Ichijo T Nader N Chrousos GP. Noncoding RNA gas5 is a growth arrest- and starvation-associated repressor of the glucocorticoid receptor. Sci Signal (2010) 3(107):ra8. 10.1126/scisignal.2000568 20124551 PMC2819218

[107] Williams GT Mourtada-Maarabouni M Farzaneh F. A critical role for non-coding RNA GAS5 in growth arrest and rapamycin inhibition in human T-lymphocytes. Biochem Soc Trans (2011) 39(2):482–6. 10.1042/BST0390482 21428924

[108] Bird A. Perceptions of epigenetics. Nature (2007) 447(7143):396–8. 10.1038/nature05913 17522671

[109] Goldberg AD Allis CD Bernstein E. Epigenetics: a landscape takes shape. Cell (2007) 128(4):635–8. 10.1016/j.cell.2007.02.006 17320500

[110] Costa FF. Non-coding RNAs, epigenetics and complexity. Gene (2008) 410(1):9–17. 10.1016/j.gene.2007.12.008 18226475

[111] Ansel KM Lee DU Rao A. An epigenetic view of helper T cell differentiation. Nat Immunol (2003) 4(7):616–23. 10.1038/ni0703-616 12830136

[112] Tripathi SK Lahesmaa R. Transcriptional and epigenetic regulation of T-helper lineage specification. Immunol Rev (2014) 261(1):62–83. 10.1111/imr.12204 25123277 PMC4255756

[113] Ansel KM Djuretic I Tanasa B Rao A. Regulation of Th2 differentiation and Il4 locus accessibility. Annu Rev Immunol (2006) 24:607–56. 10.1146/annurev.immunol.23.021704.115821 16551261

[114] Balasubramani A Mukasa R Hatton RD Weaver CT. Regulation of the Ifng locus in the context of T-lineage specification and plasticity. Immunol Rev (2010) 238(1):216–32. 10.1111/j.1600-065X.2010.00961.x 20969595 PMC3096439

[115] Chang S Aune TM. Dynamic changes in histone-methylation ‘marks’ across the locus encoding interferon-gamma during the differentiation of T helper type 2 cells. Nat Immunol (2007) 8(7):723–31. 10.1038/ni1473 17546034

[116] Ballas ZK The use of 5-azacytidine to establish constitutive interleukin 2-producing clones of the EL4 thymoma. J Immunol (1984) 133(1):7–9. 6202793

[117] Young HA Ghosh P Ye J Lederer J Lichtman A Gerard JR Differentiation of the T helper phenotypes by analysis of the methylation state of the IFN-gamma gene. J Immunol (1994) 153(8):3603–10. 7523497

[118] Bird JJ Brown DR Mullen AC Moskowitz NH Mahowald MA Sider JR Helper T cell differentiation is controlled by the cell cycle. Immunity (1998) 9(2):229–37 10.1016/S1074-7613(00)80605-6 9729043

[119] Morinobu A Kanno Y O’Shea JJ. Discrete roles for histone acetylation in human T helper 1 cell-specific gene expression. J Biol Chem (2004) 279(39):40640–6. 10.1074/jbc.M407576200 15280353

[120] Valapour M Guo J Schroeder JT Keen J Cianferoni A Casolaro V Histone deacetylation inhibits IL4 gene expression in T cells. J Allergy Clin Immunol (2002) 109(2):238–45. 10.1067/mai.2002.121145 11842291

[121] Hutchins AS Mullen AC Lee HW Sykes KJ High FA Hendrich BD Gene silencing quantitatively controls the function of a developmental trans-activator. Mol Cell (2002) 10(1):81–91. 10.1016/S1097-2765(02)00564-6 12150909

[122] Lee PP Fitzpatrick DR Beard C Jessup HK Lehar S Makar KW A critical role for Dnmt1 and DNA methylation in T cell development, function, and survival. Immunity (2001) 15(5):763–74. 10.1016/S1074-7613(01)00227-8 11728338

[123] Makar KW Perez-Melgosa M Shnyreva M Weaver WM Fitzpatrick DR Wilson CB. Active recruitment of DNA methyltransferases regulates interleukin 4 in thymocytes and T cells. Nat Immunol (2003) 4(12):1183–90. 10.1038/ni1004 14595437

[124] Yamashita M Hirahara K Shinnakasu R Hosokawa H Norikane S Kimura MY Crucial role of MLL for the maintenance of memory T helper type 2 cell responses. Immunity (2006) 24(5):611–22. 10.1016/j.immuni.2006.03.017 16713978

[125] Onodera A Yamashita M Endo Y Kuwahara M Tofukuji S Hosokawa H STAT6-mediated displacement of polycomb by trithorax complex establishes long-term maintenance of GATA3 expression in T helper type 2 cells. J Exp Med (2010) 207(11):2493–506. 10.1084/jem.20100760 20956546 PMC2964576

[126] Kimura M Koseki Y Yamashita M Watanabe N Shimizu C Katsumoto T Regulation of Th2 cell differentiation by mel-18, a mammalian polycomb group gene. Immunity (2001) 15(2):275–87 10.1016/S1074-7613(01)00182-0 11520462

[127] Yamashita M Kuwahara M Suzuki A Hirahara K Shinnaksu R Hosokawa H Bmi1 regulates memory CD4 T cell survival via repression of the Noxa gene. J Exp Med (2008) 205(5):1109–20. 10.1084/jem.20072000 18411339 PMC2373843

[128] Koyanagi M Baguet A Martens J Margueron R Jenuwein T Bix M. EZH2 and histone 3 trimethyl lysine 27 associated with Il4 and Il13 gene silencing in Th1 cells. J Biol Chem (2005) 280(36):31470–7. 10.1074/jbc.M504766200 16009709

[129] Su IH Dobenecker MW Dickinson E Oser M Basavaraj A Marqueron R Polycomb group protein ezh2 controls actin polymerization and cell signaling. Cell (2005) 121(3):425–36. 10.1016/j.cell.2005.02.029 15882624

[130] Wei G Wei L Zhu J Zang C Hu-Li J Yao Z Global mapping of H3K4me3 and H3K27me3 reveals specificity and plasticity in lineage fate determination of differentiating CD4+ T cells. Immunity (2009) 30(1):155–67. 10.1016/j.immuni.2008.12.009 19144320 PMC2722509

[131] Hirahara K Vahedi G Ghoreschi K Yang XP Nakayamada S Kanno Y Helper T-cell differentiation and plasticity: insights from epigenetics. Immunology (2011) 134(3):235–45. 10.1111/j.1365-2567.2011.03483.x 21977994 PMC3209564

[132] Bernstein BE Meissner A Lander ES The mammalian epigenome. Cell (2007) 128(4):669–81 10.1016/j.cell.2007.01.033 17320505

[133] Grogan JL Mohrs M Harmon B Lacy DA Sedat JW Locksley RM. Early transcription and silencing of cytokine genes underlie polarization of T helper cell subsets. Immunity (2001) 14(3):205–15. 10.1016/S1074-7613(01)00103-0 11290331

[134] Mullen AC High FA Hutchins AS Lee HW Villarino AV Livingston DM Role of T-bet in commitment of TH1 cells before IL-12-dependent selection. Science (2001) 292(5523):1907–10. 10.1126/science.1059835 11397944

[135] Panzer M Sitte S Wirth S Drexler I Sparwasser T Voehringer D. Rapid in vivo conversion of effector T cells into Th2 cells during helminth infection. J Immunol (2012) 188(2):615–23. 10.4049/jimmunol.1101164 22156341

[136] Peine M Rausch S Helmstetter C Fröhlich A Hegazy AN Kühl AA Stable T-bet(+)GATA-3(+) Th1/Th2 hybrid cells arise *in vivo*, can develop directly from naive precursors, and limit immunopathologic inflammation. PLoS Biol (2013) 11(8):e1001633. 10.1371/journal.pbio.1001633 23976880 PMC3747991

[137] Zhao J Ohsumi TK Kung JT Ogawa Y Grau DJ Sarma K Genome-wide identification of polycomb-associated RNAs by RIP-seq. Mol Cell (2010) 40(6):939–53. 10.1016/j.molcel.2010.12.011 21172659 PMC3021903

[138] Tsai MC Manor O Wan Y Mosammaparast N Wang JK Lan F Long noncoding RNA as modular scaffold of histone modification complexes. Science (2010) 329(5992):689–93. 10.1126/science.1192002 20616235 PMC2967777

[139] Guttman M Donaghey J Carey BW Garber M Grenier JK Munson G lincRNAs act in the circuitry controlling pluripotency and differentiation. Nature (2011) 477(7364):295–300. 10.1038/nature10398 21874018 PMC3175327

[140] Schuettengruber B Ganapathi M Leblanc B Portoso M Jaschek R Tolhuis B Functional anatomy of polycomb and trithorax chromatin landscapes in *Drosophila* embryos. PLoS Biol (2009) 7(1):e1000013. 10.1371/journal.pbio.1000013 19143474 PMC2621266

[141] Kassis JA Brown JL Polycomb group response elements in *Drosophila* and vertebrates. Adv Genet (2013) 81:83–118 10.1016/B978-0-12-407677-8.00003-8 23419717 PMC4157523

[142] Bernstein E Allis CD RNA meets chromatin. Genes Dev (2005) 19(14):1635–55 10.1101/gad.1324305 16024654

[143] Jeffery L Nakielny S. Components of the DNA methylation system of chromatin control are RNA-binding proteins. J Biol Chem (2004) 279(47):49479–87. 10.1074/jbc.M409070200 15342650

[144] Hiragami-Hamada K Fischle W. RNAs – physical and functional modulators of chromatin reader proteins. Biochim Biophys Acta (2014) 1839(8):737–42. 10.1016/j.bbagrm.2014.03.015 24704208

[145] Zhao J Sun BK Erwin JA Song J-J Lee JT. Polycomb proteins targeted by a short repeat RNA to the mouse X chromosome. Science (2008) 322(5902):750–6. 10.1126/science.1163045 18974356 PMC2748911

[146] Pandey RR Mondal T Mohammad F Enroth S Redrup L Komorowski J Kcnq1ot1 antisense noncoding RNA mediates lineage-specific transcriptional silencing through chromatin-level regulation. Mol Cell (2008) 32(2):232–46. 10.1016/j.molcel.2008.08.022 18951091

[147] Yang L Lin C Liu W Zhang J Ohgi KA Grinstein JD ncRNA- and Pc2 methylation-dependent gene relocation between nuclear structures mediates gene activation programs. Cell (2011) 147(4):773–88. 10.1016/j.cell.2011.08.054 22078878 PMC3297197

[148] Chalei V Sansom SN Kong L Lee S Montiel JF Vance KW The long non-coding RNA Dali is an epigenetic regulator of neural differentiation. Elife (2014) 3:e04530. 10.7554/eLife.04530 25415054 PMC4383022

[149] Steffen PA Ringrose L. What are memories made of? How polycomb and trithorax proteins mediate epigenetic memory. Nat Rev Mol Cell Biol (2014) 15(5):340–56. 10.1038/nrm3789 24755934

[150] Arab K Park YJ Lindroth AM Schafer A Oakes C Weichenhan D Long noncoding RNA TARID directs demethylation and activation of the tumor suppressor TCF21 via GADD45A. Mol Cell (2014) 55(4):604–14. 10.1016/j.molcel.2014.06.031 25087872

[151] Iyer LM Abhiman S Aravind L. Natural history of eukaryotic DNA methylation systems. Prog Mol Biol Transl Sci (2011) 101:25–104. 10.1016/B978-0-12-387685-0.00002-0 21507349

[152] Ponnaluri VK Maciejewski JP Mukherji M. A mechanistic overview of TET-mediated 5-methylcytosine oxidation. Biochem Biophys Res Commun (2013) 436(2):115–20. 10.1016/j.bbrc.2013.05.077 23727577

[153] Lee JT. Epigenetic regulation by long noncoding RNAs. Science (2012) 338(6113):1435–9. 10.1126/science.1231776 23239728

[154] Rinn JL Kertesz M Wang JK Squazzo SL Xu X Brugmann SA Functional demarcation of active and silent chromatin domains in human HOX loci by noncoding RNAs. Cell (2007) 129(7):1311–23. 10.1016/j.cell.2007.05.022 17604720 PMC2084369

[155] Cremer T Cremer C Chromosome territories, nuclear architecture and gene regulation in mammalian cells. Nat Rev Genet (2001) 2(4):292–301 10.1038/35066075 11283701

[156] Sleeman JE Trinkle-Mulcahy L. Nuclear bodies: new insights into assembly/dynamics and disease relevance. Curr Opin Cell Biol (2014) 28(0):76–83. 10.1016/j.ceb.2014.03.004 24704702

[157] Reddy KL Zullo JM Bertolino E Singh H. Transcriptional repression mediated by repositioning of genes to the nuclear lamina. Nature (2008) 452(7184):243–7. 10.1038/nature06727 18272965

[158] Kumaran RI Thakar R Spector DL Chromatin dynamics and gene positioning. Cell (2008) 132(6):929–34 10.1016/j.cell.2008.03.004 18358806 PMC2898133

[159] Kosak ST Skok JA Medina KL Riblet R Le Beau MM Fisher AG Subnuclear compartmentalization of immunoglobulin loci during lymphocyte development. Science (2002) 296(5565):158–62. 10.1126/science.1068768 11935030

[160] Yao H Brick K Evrard Y Xiao T Camerini-Otero RD Felsenfeld G. Mediation of CTCF transcriptional insulation by DEAD-box RNA-binding protein p68 and steroid receptor RNA activator SRA. Genes Dev (2010) 24(22):2543–55. 10.1101/gad.1967810 20966046 PMC2975930

[161] Xiang J-F Yin Q-F Chen T Zhang Y Zhang X-O Wu Z Human colorectal cancer-specific CCAT1-L lncRNA regulates long-range chromatin interactions at the MYC locus. Cell Res (2014) 24(5):513–31. 10.1038/cr.2014.35 24662484 PMC4011346

[162] Hacisuleyman E Goff LA Trapnell C Williams A Henao-Mejia J Sun L Topological organization of multichromosomal regions by the long intergenic noncoding RNA Firre. Nat Struct Mol Biol (2014) 21(2):198–206. 10.1038/nsmb.2764 24463464 PMC3950333

[163] Lai F Orom UA Cesaroni M Beringer M Taatjes DJ Blobel GA Activating RNAs associate with mediator to enhance chromatin architecture and transcription. Nature (2013) 494(7438):497–501. 10.1038/nature11884 23417068 PMC4109059

[164] Orom UA Derrien T Beringer M Gumireddy K Gardini A Bussotti G Long noncoding RNAs with enhancer-like function in human cells. Cell (2010) 143(1):46–58. 10.1016/j.cell.2010.09.001 20887892 PMC4108080

[165] Krivega I Dean A. Enhancer and promoter interactions-long distance calls. Curr Opin Genet Dev (2012) 22(2):79–85. 10.1016/j.gde.2011.11.001 22169023 PMC3342482

[166] Kagey MH Newman JJ Bilodeau S Zhan Y Orlando DA van Berkum NL Mediator and cohesin connect gene expression and chromatin architecture. Nature (2010) 467(7314):430–5. 10.1038/nature09380 20720539 PMC2953795

[167] O’Shea JJ Paul WE. Mechanisms underlying lineage commitment and plasticity of helper CD4+ T cells. Science (2010) 327(5969):1098–102. 10.1126/science.1178334 20185720 PMC2997673

[168] Geginat J Paroni M Maglie S Alfen JS Kastirr I Gruarin P Plasticity of human CD4 T cell subsets. Front Immunol (2014) 5:630. 10.3389/fimmu.2014.00630 25566245 PMC4267263

[169] Hedrich CM Bream JH. Cell type-specific regulation of IL-10 expression in inflammation and disease. Immunol Res (2010) 47(1–3):185–206. 10.1007/s12026-009-8150-5 20087682 PMC2892196

[170] Chen Z Tato CM Muul L Laurence A O’Shea JJ. Distinct regulation of interleukin-17 in human T helper lymphocytes. Arthritis Rheum (2007) 56(9):2936–46. 10.1002/art.22866 17763419 PMC2323677

[171] Wilson NJ Boniface K Chan JR McKenzie BS Blumenschein WM Mattson JD Development, cytokine profile and function of human interleukin 17-producing helper T cells. Nat Immunol (2007) 8(9):950–7. 10.1038/ni1497 17676044

[172] Zhang F Meng G Strober W. Interactions among the transcription factors Runx1, RORgammat and Foxp3 regulate the differentiation of interleukin 17-producing T cells. Nat Immunol (2008) 9(11):1297–306. 10.1038/ni.1663 18849990 PMC4778724

[173] Klunker S Chong MM Mantel PY Palomares O Bassin C Ziegler M Transcription factors RUNX1 and RUNX3 in the induction and suppressive function of Foxp3+ inducible regulatory T cells. J Exp Med (2009) 206(12):2701–15. 10.1084/jem.20090596 19917773 PMC2806624

[174] Li L Patsoukis N Petkova V Boussiotis VA. Runx1 and Runx3 are involved in the generation and function of highly suppressive IL-17-producing T regulatory cells. PLoS One (2012) 7(9):e45115. 10.1371/journal.pone.0045115 22984619 PMC3440330

[175] Chung Y Tanaka S Chu F Nurieva RI Martinez GJ Rawal S Follicular regulatory T cells expressing Foxp3 and Bcl-6 suppress germinal center reactions. Nat Med (2011) 17(8):983–8. 10.1038/nm.2426 21785430 PMC3151340

[176] Tsuji M Komatsu N Kawamoto S Suzuki K Kanagawa O Honjo T Preferential generation of follicular B helper T cells from Foxp3+ T cells in gut Peyer’s patches. Science (2009) 323(5920):1488–92. 10.1126/science.1169152 19286559

[177] Rossi RL Rossetti G Wenandy L Curti S Ripamonti A Bonnal RJP Distinct microRNA signatures in human lymphocyte subsets and enforcement of the naive state in CD4+ T cells by the microRNA miR-125b. Nat Immunol (2011) 12(8):796–803. 10.1038/ni.2057 21706005

[178] Turner M Galloway A Vigorito E. Noncoding RNA and its associated proteins as regulatory elements of the immune system. Nat Immunol (2014) 15(6):484–91. 10.1038/ni.2887 24840979

[179] Barabasi A-L Oltvai ZN Network biology: understanding the cell’s functional organization. Nat Rev Genet (2004) 5(2):101–13 10.1038/nrg1272 14735121

[180] Zhang B Arun G Mao Yuntao S Lazar Z Hung G Bhattacharjee G The lncRNA Malat1 is dispensable for mouse development but its transcription plays a cis-regulatory role in the adult. Cell Rep (2013) 2(1):111–23. 10.1016/j.celrep.2012.06.003 22840402 PMC3408587

[181] Necsulea A Kaessmann H. Evolutionary dynamics of coding and non-coding transcriptomes. Nat Rev Genet (2014) 15(11):734–48. 10.1038/nrg3802 25297727

[182] Pollard KS Salama SR King B Kern AD Dreszer T Katzman S Forces shaping the fastest evolving regions in the human genome. PLoS Genet (2006) 2(10):e168. 10.1371/journal.pgen.0020168 17040131 PMC1599772

[183] Schorderet P Duboule D. Structural and functional differences in the long non-coding RNA hotair in mouse and human. PLoS Genet (2011) 7(5):e1002071. 10.1371/journal.pgen.1002071 21637793 PMC3102750

[184] Gerstein MB Rozowsky J Yan KK Wang D Cheng C Brown JB Comparative analysis of the transcriptome across distant species. Nature (2014) 512(7515):445–8. 10.1038/nature13424 25164755 PMC4155737

[185] On T Xiong X Pu S Turinsky A Gong Y Emili A The evolutionary landscape of the chromatin modification machinery reveals lineage specific gains, expansions, and losses. Proteins (2010) 78(9):2075–89. 10.1002/prot.22723 20455264

[186] Hrdlickova B Kumar V Kanduri K Zhernakova D Tripathi S Karjalainen J Expression profiles of long non-coding RNAs located in autoimmune disease-associated regions reveal immune cell-type specificity. Genome Med (2014) 6(10):88. 10.1186/s13073-014-0088-0 25419237 PMC4240855

[187] Xia F Dong F Yang Y Huang A Chen S Sun D Dynamic transcription of long non-coding RNA genes during CD4+ T cell development and activation. PLoS One (2014) 9(7):e101588. 10.1371/journal.pone.0101588 25003630 PMC4086894

[188] Aoki K Harashima A Sano M Yokoi T Nakamura S Kibata M A thymus-specific noncoding RNA, Thy-ncR1, is a cytoplasmic riboregulator of MFAP4 mRNA in immature T-cell lines. BMC Mol Biol (2010) 11(1):99. 10.1186/1471-2199-11-99 21162727 PMC3023731

[189] Hu G Tang Q Sharma S Yu F Escobar TM Muljo SA Expression and regulation of intergenic long noncoding RNAs during T cell development and differentiation. Nat Immunol (2013) 14(11):1190–8. 10.1038/ni.2712 24056746 PMC3805781

[190] Kimura T Jiang S Nishizawa M Yoshigai E Hashimoto I Nishikawa M Stabilization of human interferon-alpha1 mRNA by its antisense RNA. Cell Mol Life Sci (2013) 70(8):1451–67. 10.1007/s00018-012-1216-x 23224365 PMC3607724

[191] Yan M-D Hong C-C Lai G-M Cheng A-L Lin Y-W Chuang S-E. Identification and characterization of a novel gene Saf transcribed from the opposite strand of Fas. Hum Mol Genet (2005) 14(11):1465–74. 10.1093/hmg/ddi156 15829500

[192] Sehgal L Mathur R Braun FK Wise JF Berkova Z Neelapu S FAS-antisense 1 lncRNA and production of soluble versus membrane Fas in B-cell lymphoma. Leukemia (2014) 28(12):2376–87. 10.1038/leu.2014.126 24811343 PMC5827933

[193] Haasch D Chen YW Reilly RM Chiou XG Koterski S Smith ML T cell activation induces a noncoding RNA transcript sensitive to inhibition by immunosuppressant drugs and encoded by the proto-oncogene, BIC. Cell Immunol (2002) 217(1–2):78–86. 10.1016/S0008-8749(02)00506-3 12426003

[194] Liu AY Torchia BS Migeon BR Siliciano RF. The human NTT gene: identification of a novel 17-kb noncoding nuclear RNA expressed in activated CD4+ T cells. Genomics (1997) 39(2):171–84. 10.1006/geno.1996.4463 9027504

[195] Abarrategui I Krangel MS. Regulation of T cell receptor-alpha gene recombination by transcription. Nat Immunol (2006) 7(10):1109–15. 10.1038/ni1379 16936730

[196] Collier SP Collins PL Williams CL Boothby MR Aune TM. Cutting edge: influence of Tmevpg1, a long intergenic noncoding RNA, on the expression of Ifng by Th1 cells. J Immunol (2012) 189(5):2084–8. 10.4049/jimmunol.1200774 22851706 PMC3424368

[197] Gomez JA Wapinski OL Yang YW Bureau JF Gopinath S Monack DM The NeST long ncRNA controls microbial susceptibility and epigenetic activation of the interferon-gamma locus. Cell (2013) 152(4):743–54. 10.1016/j.cell.2013.01.015 23415224 PMC3577098

[198] Zhang H Nestor CE Zhao S Lentini A Bohle B Benson M Profiling of human CD4+ T-cell subsets identifies the TH2-specific noncoding RNA GATA3-AS1. J Allergy Clin Immunol (2013) 132(4):1005–8 10.1016/j.jaci.2013.05.033 23870669

[199] Seok J Warren HS Cuenca AG Mindrinos MN Baker HV Xu W Genomic responses in mouse models poorly mimic human inflammatory diseases. Proc Natl Acad Sci U S A (2013) 110(9):3507–12. 10.1073/pnas.1222878110 23401516 PMC3587220

[200] Takao K Miyakawa T. Genomic responses in mouse models greatly mimic human inflammatory diseases. Proc Natl Acad Sci U S A (2015) 112(4):1167–72. 10.1073/pnas.1401965111 25092317 PMC4313832

[201] Tuomela S Lahesmaa R. Early T helper cell programming of gene expression in human. Semin Immunol (2013) 25(4):282–90. 10.1016/j.smim.2013.10.013 24246225

[202] Necsulea A Soumillon M Warnefors M Liechti A Daish T Zeller U The evolution of lncRNA repertoires and expression patterns in tetrapods. Nature (2014) 505(7485):635–40. 10.1038/nature12943 24463510

[203] Nesterova TB Slobodyanyuk SY Elisaphenko EA Shevchenko AI Johnston C Pavlova ME Characterization of the genomic Xist locus in rodents reveals conservation of overall gene structure and tandem repeats but rapid evolution of unique sequence. Genome Res (2001) 11(5):833–49. 10.1101/gr.174901 11337478 PMC311126

[204] Cabili MN Trapnell C Goff L Koziol M Tazon-Vega B Regev A Integrative annotation of human large intergenic noncoding RNAs reveals global properties and specific subclasses. Genes Dev (2011) 25(18):1915–27. 10.1101/gad.17446611 21890647 PMC3185964

[205] Church DM Goodstadt L Hillier LW Zody MC Goldstein S She X Lineage-specific biology revealed by a finished genome assembly of the mouse. PLoS Biol (2009) 7(5):e1000112. 10.1371/journal.pbio.1000112 19468303 PMC2680341

[206] Pang KC Frith MC Mattick JS. Rapid evolution of noncoding RNAs: lack of conservation does not mean lack of function. Trends Genet (2006) 22(1):1–5. 10.1016/j.tig.2005.10.003 16290135

[207] Carninci P Kasukawa T Katayama S Gough J Frith MC Maeda N The transcriptional landscape of the mammalian genome. Science (2005) 309(5740):1559–63. 10.1126/science.1112014 16141072

[208] Ulitsky I Shkumatava A Jan Calvin H Sive H Bartel David P. Conserved function of lincRNAs in vertebrate embryonic development despite rapid sequence evolution. Cell (2011) 147(7):1537–50. 10.1016/j.cell.2011.11.055 22196729 PMC3376356

[209] He S Liu S Zhu H. The sequence, structure and evolutionary features of HOTAIR in mammals. BMC Evol Biol (2011) 11:102. 10.1186/1471-2148-11-102 21496275 PMC3103462

[210] Zhang X Lian Z Padden C Gerstein MB Rozowsky J Snyder M A myelopoiesis-associated regulatory intergenic noncoding RNA transcript within the human HOXA cluster. Blood (2009) 113(11):2526–34. 10.1182/blood-2008-06-162164 19144990 PMC2656274

[211] Wright PW Huehn A Cichocki F Li H Sharma N Dang H Identification of a KIR antisense lncRNA expressed by progenitor cells. Genes Immun (2013) 14(7):427–33. 10.1038/gene.2013.36 23863987 PMC3808466

[212] Wang P Xue Y Han Y Lin L Wu C Xu S The STAT3-binding long noncoding RNA lnc-DC controls human dendritic cell differentiation. Science (2014) 344(6181):310–3. 10.1126/science.1251456 24744378

[213] Xing Z Lin A Li C Liang K Wang S Liu Y lncRNA directs cooperative epigenetic regulation downstream of chemokine signals. Cell (2014) 159(5):1110–25. 10.1016/j.cell.2014.10.013 25416949 PMC4266991

[214] Carpenter S Aiello D Atianand MK Ricci EP Gandhi P Hall LL A long noncoding RNA mediates both activation and repression of immune response genes. Science (2013) 341(6147):789–92. 10.1126/science.1240925 23907535 PMC4376668

[215] Rapicavoli NA Qu K Zhang J Mikhail M Laberge R-M Chang HY. A mammalian pseudogene lncRNA at the interface of inflammation and anti-inflammatory therapeutics. Elife (2013) 2:e00762. 10.7554/eLife.00762 23898399 PMC3721235

[216] Li Z Chao T-C Chang K-Y Lin N Patil VS Shimizu C The long noncoding RNA THRIL regulates TNFα expression through its interaction with hnRNPL. Proc Natl Acad Sci U S A (2014) 111(3):1002–7. 10.1073/pnas.1313768111 24371310 PMC3903238

[217] Mosmann TR Cherwinski H Bond MW Giedlin MA Coffman RL Two types of murine helper T cell clone. I. Definition according to profiles of lymphokine activities and secreted proteins. J Immunol (1986) 136(7):2348–57. 2419430

[218] Chang S Aune TM. Histone hyperacetylated domains across the Ifng gene region in natural killer cells and T cells. Proc Natl Acad Sci U S A (2005) 102(47):17095–100. 10.1073/pnas.0502129102 16286661 PMC1283154

[219] Zhou W Chang S Aune TM. Long-range histone acetylation of the Ifng gene is an essential feature of T cell differentiation. Proc Natl Acad Sci U S A (2004) 101(8):2440–5. 10.1073/pnas.0306002101 14983028 PMC356969

[220] Ho IC Lo D Glimcher LH. c-maf promotes T helper cell type 2 (Th2) and attenuates Th1 differentiation by both interleukin 4-dependent and -independent mechanisms. J Exp Med (1998) 188(10):1859–66. 10.1084/jem.188.10.1859 9815263 PMC2212398

[221] Liu X Nurieva RI Dong C. Transcriptional regulation of follicular T-helper (Tfh) cells. Immunol Rev (2013) 252(1):139–45. 10.1111/imr.12040 23405901 PMC3579502

[222] Sato K Miyoshi F Yokota K Araki Y Asanuma Y Akiyama Y Marked induction of c-Maf protein during Th17 cell differentiation and its implication in memory Th cell development. J Biol Chem (2011) 286(17):14963–71. 10.1074/jbc.M111.218867 21402704 PMC3083235

[223] Gupta RA Shah N Wang KC Kim J Horlings HM Wong DJ Long non-coding RNA HOTAIR reprograms chromatin state to promote cancer metastasis. Nature (2010) 464(7291):1071–6. 10.1038/nature08975 20393566 PMC3049919

